# Current knowledge of the implication of lipid mediators in psoriasis

**DOI:** 10.3389/fimmu.2022.961107

**Published:** 2022-08-26

**Authors:** Mélissa Simard, Sophie Morin, Zainab Ridha, Roxane Pouliot

**Affiliations:** ^1^ Centre de Recherche en Organogénèse Expérimentale de l’Université Laval/Laboratoire d’Organogénèse EXpérimentale (LOEX), Axe Médecine Régénératrice, Centre de Recherche du Centre Hospitalier Universitaire (CHU) de Québec, Québec, QC, Canada; ^2^ Faculté de Pharmacie, Université Laval, Québec, QC, Canada

**Keywords:** psoriasis, lipid mediator, skin, polyunsaturated fatty acids, inflammation

## Abstract

The skin is an organ involved in several biological processes essential to the proper functioning of the organism. One of these essential biological functions of the skin is its barrier function, mediated notably by the lipids of the stratum corneum, and which prevents both penetration from external aggression, and transepidermal water loss. Bioactive lipid mediators derived from polyunsaturated fatty acids (PUFAs) constitute a complex bioactive lipid network greatly involved in skin homeostasis. Bioactive lipid mediators derived from n-3 and n-6 PUFAs have well-documented anti- and pro-inflammatory properties and are recognized as playing numerous and complex roles in the behavior of diverse skin diseases, including psoriasis. Psoriasis is an inflammatory autoimmune disease with many comorbidities and is associated with enhanced levels of pro-inflammatory lipid mediators. Studies have shown that a high intake of n-3 PUFAs can influence the development and progression of psoriasis, mainly by reducing the severity and frequency of psoriatic plaques. Herein, we provide an overview of the differential effects of n-3 and n-6 PUFA lipid mediators, including prostanoids, hydroxy-fatty acids, leukotrienes, specialized pro-resolving mediators, *N*-acylethanolamines, monoacylglycerols and endocannabinoids. This review summarizes current findings on lipid mediators playing a role in the skin and their potential as therapeutic targets for psoriatic patients.

## Introduction

Lipid metabolism is currently a major research interest as lipids play crucial roles in all biological mechanisms, including the formation and functioning of the skin barrier. Interest in the metabolism of n-3 and n-6 polyunsaturated fatty acids (PUFAs) was first shown by Burr and Burr in the 1920s, in studies investigating the effects of different PUFAs on rodent health through diet supplementation (1). These studies led to the identification of two polyunsaturated fatty acids, linoleic acid (LA, n-6 PUFA) and α-linolenic acid (ALA, n-3 PUFA), which cannot be synthesized by mammalian organism and whose presence in the body is therefore dependent on food intakes. It was later shown that PUFAs and PUFA-derived bioactive lipid mediators play important roles in the regulation of biological processes by mediating inflammatory responses. Bioactive lipid mediators can be found throughout the body, and each tissue has a characteristic bioactive lipid mediator profile. This specificity depends on the expression, activity, and affinity of different PUFAs, and of the proteins involved in the biosynthesis of bioactive lipid mediators, which vary among tissues (2). Moreover, the bioactive lipid mediator profile of an individual is largely affected by their nutritional status (3, 4).

Studies on the Inuit paradox in the 1970s highlighted the diverse benefits of high n-3 PUFA consumption on the risk of developing cardiovascular diseases (5). Since then, many clinical studies have investigated the effects of diet supplementation with n-3 PUFAs on the risk of developing cancer, atherosclerosis and inflammatory skin diseases, such as psoriasis (6–8). In parallel, many bioactive lipid mediators derived from n-6 PUFAs were being discovered and associated with leukocyte chemoattractant and pro-inflammatory functions (9, 10). Following these studies, n-3 and n-6 PUFA-derived bioactive lipid mediators were recognized for their unique and complementary inflammatory properties (11). Those derived from n-3 PUFAs were primarily known for their anti-inflammatory, beneficial, and protective properties, in contrast to those derived from n-6 PUFAs, which are better known for their pro-inflammatory properties. The n-3 and n-6 PUFAs are metabolized by the same enzymes; consequently, their transformation is regulated by substrate competition (12). The duality of the effects of the two PUFA families led to a concept widely discussed in the literature, namely the balance between n-3 and n-6 PUFAs (13, 14). However, the effects of PUFAs were shown to be much more extensive and complex. For instance, both n-3 and n-6 PUFA-derived bioactive lipid mediators can have pro- or anti-inflammatory effects, leading to contradictory conclusions. Indeed, n-3 and n-6 PUFAs follow multiple complex metabolic pathways, making it difficult to establish their precise mode of action (15). At the beginning of the 21^st^ century, a new concept emerged, reporting that the acute inflammatory reaction progresses in two phases: the initiation and the resolution of inflammation (16). Numerous studies appear to convincingly show that lipid mediators are produced in a sequential and organized manner to regulate the inflammatory response (17–21). Lipid mediators derived from n-6 PUFAs, such as prostaglandin E_2_ (PGE_2_) and leukotriene B_4_ (LTB_4_), would be produced during the initiation phase in order to promote in particular the recruitment and the adhesion of immune cells, as well as the dilation of blood vessels (22). The resolution phase would be mediated by the production of lipid mediators derived from n-3 PUFAs and more specifically by specialized pro-resolving mediators (SPMs) (23, 24).

Psoriasis is an inflammatory skin disease in which a dysregulation in lipid mediators derived from n-6 PUFAs has been reported (25, 26). Hence, n-3 PUFAs have been investigated as potential treatment options in various studies. While the results of these studies are varied, the evidence seems to favor a beneficial effect of n-3 PUFAs on psoriasis. This manuscript reviews current knowledge of the implication of n-3 and n-6 PUFAs, as well as their related lipid mediators, in psoriasis and their potential therapeutic effects on this skin condition.

## Skin physiology

The skin is a crucial organ that is essential to human survival. Among its multiple roles, the barrier function is undeniably important: the skin barrier prevents the bodily penetration of external substances while maintaining the hydration of the body by preventing water loss (27). The structure of the skin is divided into three layers: hypodermis, dermis, and epidermis. The hypodermis is the deepest layer, mainly constituted of adipocytes playing an important role in thermoregulation and maintaining the energy reserve of the body. The dermis, the middle layer, is a connective tissue composed of fibroblasts surrounded by a rich extracellular matrix conferring suppleness and elasticity to the skin (28). It contains blood vessels, nerves, and skin appendages including sweat glands and hair follicles. This layer supports and nourishes the epidermal cells by diffusion (28, 29). The epidermis is the skin’s most superficial layer, a squamous epithelium mainly constituted of keratinocytes and in which other cell types are incorporated including Langerhans cells, Merkel cells, and melanocytes (28). Skin immunity is crucial for protecting the body from harmful external aggressions. The immune system of the skin involves both innate and adaptive immunity. The innate immunity includes two lines of defense, one provided by epithelial tissues and the other provided by specific immune cells, including neutrophils, monocytes, macrophages, and natural killer cells (30). In fact, keratinocytes, along with neutrophils, modulate the immune status of the skin by producing large quantities of antimicrobial peptides (AMPs), including psoriasin (S100A7), calgranulin A (S100A8), B (S100A9), β-defensins and cathelicidin (CAMP) (31, 32). Subsequently, under the influence of inflammatory stimuli, cytotoxic T cells (CD8+), which eliminate infected and cancerous cells, and helper T (Th) cells (CD4+), which coordinate humoral immunity and stimulate B cells, can infiltrate the skin (33). The dermis permanently harbors immune cells, including dendritic cells and memory T cells (34).

The constant renewal of the epidermis includes the proliferation, differentiation, and elimination of keratinocytes by desquamation, with a cycle lasting approximately 28 days. The morphological, structural, and protein changes observed during this process permit the division of the epidermis into four layers, namely the stratum basal, stratum spinosum, stratum granulosum, and stratum corneum (35). The keratinocytes of the stratum basal proliferate to ensure the renewal of the epidermis (28). As the cells proliferate, they migrate towards the stratum spinosum, where the differentiation of the keratinocytes is induced through the action of desmosomes linking the cells together. The differentiation of epidermal keratinocytes is a complex but perfectly orchestrated process, leading to cell death and the formation of a semi-permeable barrier: the stratum corneum (35, 36). This process causes drastic changes such as the complete flattening of the cells and the degradation of their organelles and nuclei, ending in the formation of corneocytes (35). The stratum corneum is organized in a brick-and-mortar pattern, with the bricks being the corneocytes and the mortar the lipid matrix between the cells, thus providing a tight structure. The lipid matrix alone corresponds to 10% of the weight of the stratum corneum, and is composed of 45% ceramides, 30% cholesterol, and 15% free fatty acids (37). The cells of the stratum corneum eventually desquamate (38).

**Figure 1:**
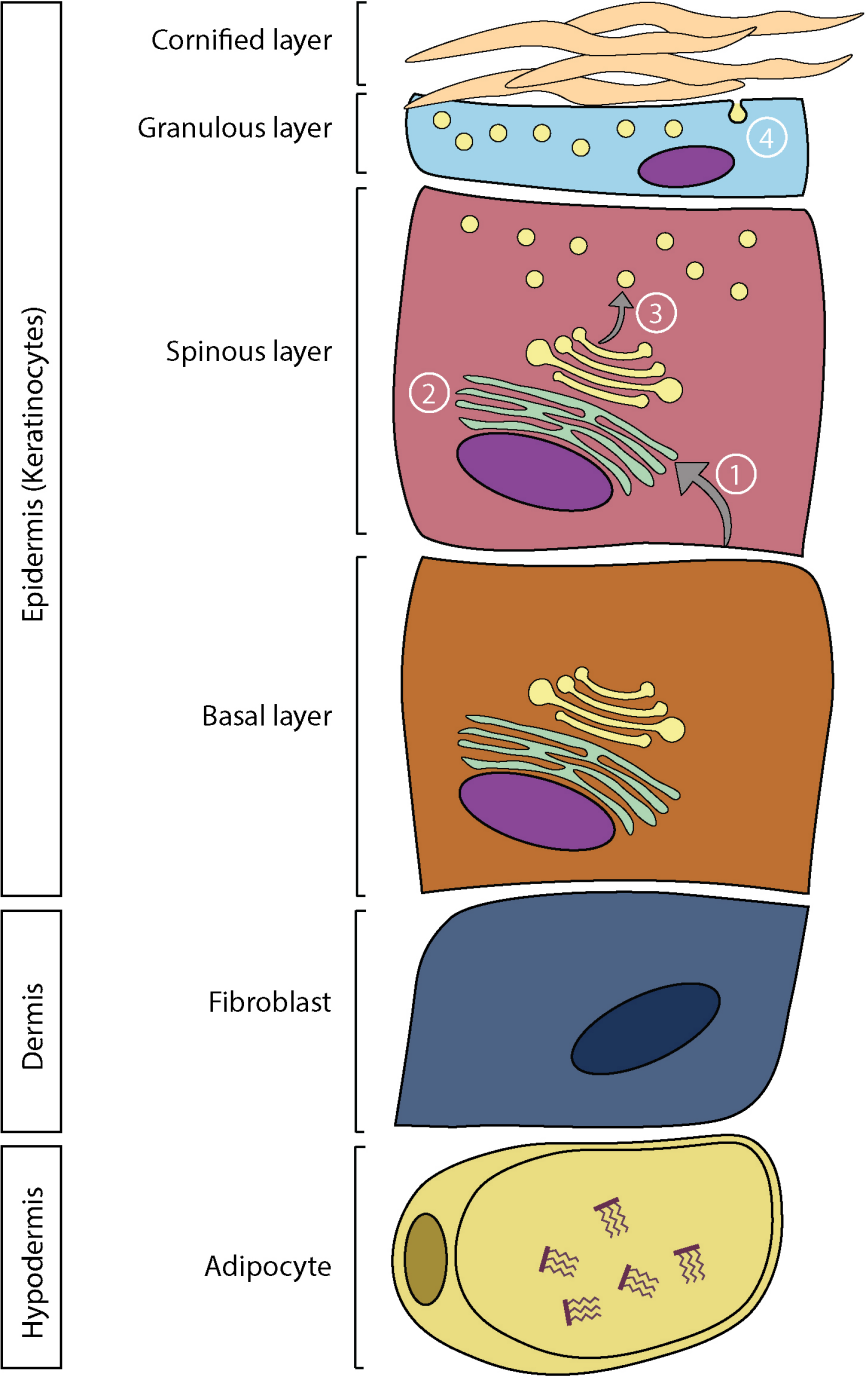
Skin structure and lipid metabolism. In the epidermis: 1) Fatty acids are hydrolyzed from phospholipids and transported in the endoplasmic reticulum. 2) Synthesis of more complex lipids. 3) Storage of lipids in lamellar bodies. 4) Extrusion of lamellar bodies and organization of the lipid matrix.

The epidermis has an important lipid metabolism, which is modulated during epidermal differentiation to ensure the production of the stratum corneum lipid matrix (39). The basal layer of the epidermis has a high rate of phospholipid biosynthesis due to the cell division process, which requires the formation of new cell membranes (40). Phospholipids account for 45% of lipids in the basal and spinous layers, 25% in the stratum granulosum, and less than 5% in the stratum corneum (28). In the spinous layer, fatty acids from cell membrane phospholipids are hydrolyzed and transported to the endoplasmic reticulum (41). They are then used for the synthesis of more complex lipids, including ceramides and triglycerides (39). Cholesterol is also synthesized *de novo* in this layer (42). Newly formed lipids are subsequently stored in vesicles produced by the Golgi apparatus, called lamellar bodies. Lamellar bodies are 0.3 mm ovoid secretory organelles, containing mostly lipids (phospholipids, cholesterol, glucosylceramides, and sphingomyelins) and several proteins (43). These proteins consist mainly of glucosidase, sphingomyelinase, secretory phospholipase A2 (sPLA2) as well as several neutral and acidic lipases, kallikreins 7 and 8, cathepsin D, inhibitory proteases, caveolin-1, and corneodesmosins (43). During cell differentiation, there is an accumulation of lamellar bodies in keratinocytes up to the granular layer where they are finally extruded, releasing their lipid contents between stratum corneum cells (39). Following their secretion, lipids are subsequently metabolized by co-secreted proteins (**Figure 1**) (43). Phospholipids are hydrolyzed by phospholipase A_2_ (PLA_2_) to generate free fatty acids for the stratum corneum lipid matrix as well as glycerol. This glycerol is responsible for the hydration of the stratum corneum (43). The elongation of fatty acids by elongases (ELOVL) is a crucial step in epidermal differentiation, since it allows the generation of free fatty acids with very long chains, which are characteristic of the stratum corneum (44, 45). Additionally, a group of ω-hydroxy-ceramides containing a LA moiety, the acylceramides, are particularly important in the proper organization of the lipid matrix. Since they are covalently bound to the proteins of the cornified envelope, they serve as a scaffold for the other lipids, which allows an optimal bilamellar organization of the lipid matrix (46). Eventually, cholesterol sulfate is converted to cholesterol which allows the desquamation of corneocytes (43).

### n-3 and n-6 PUFA metabolism

**Figure 2:**
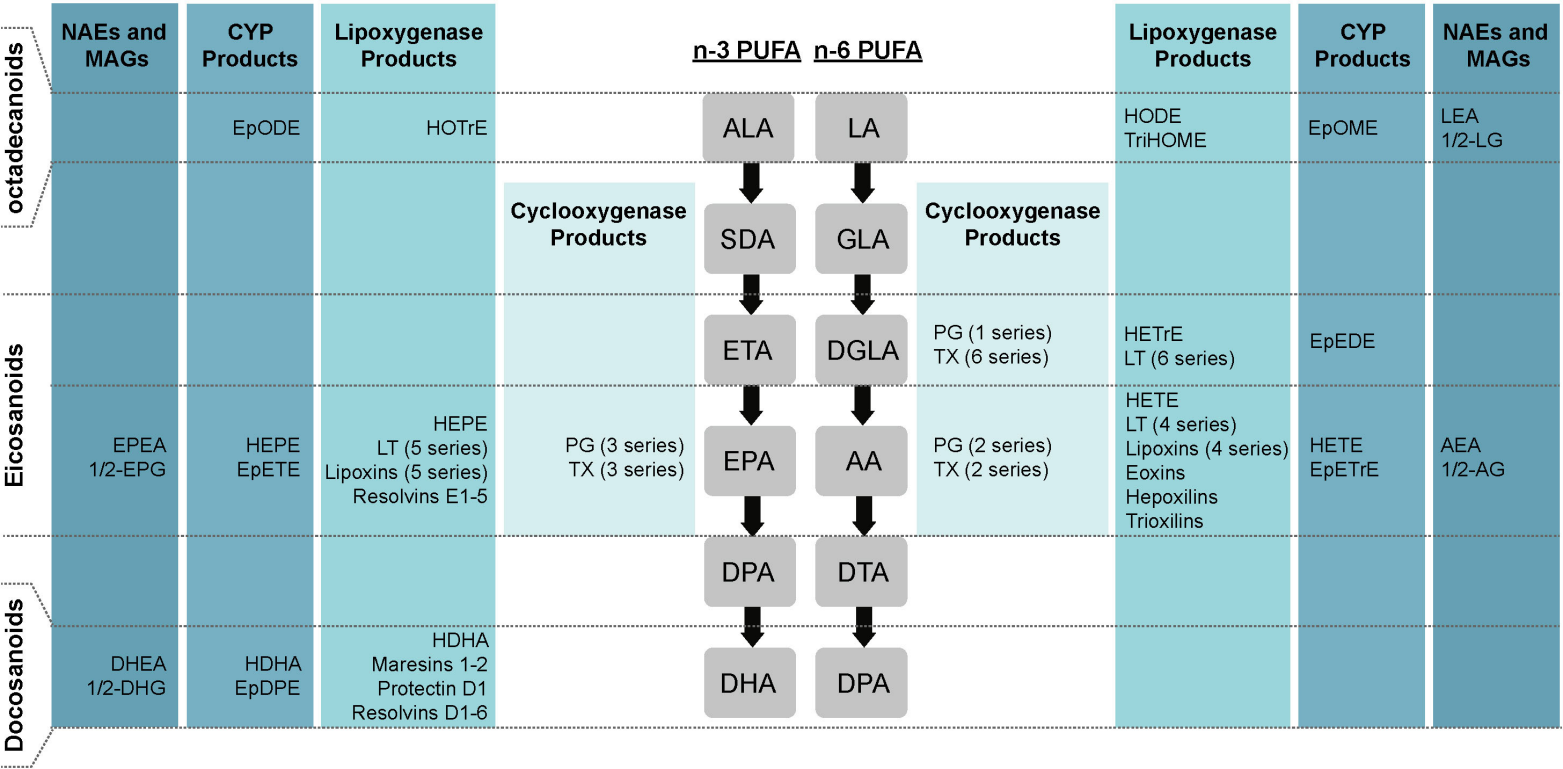
Schematic representation of different types of lipid mediators. Lipid mediators derived from ALA, EPA, DHA, LA, DGLA and AA. AA, arachidonic acid; ALA, alpha-linolenic acid; CYP, P450 cytochrome; DGLA, dihomo-gamma-linolenic acid; DHA, docosahexaenoic acid; DPA, docosapentaenoic acid; DTA, docosatetraenoic acid; EPA, eicosapentaenoic acid; EpDPE, epoxy-docosapentaenoic acid; EpEDE, epoxy-eicosadienoic acid; EpETE, epoxy-ecosatetraenoic acid; EpETrE, epoxy-eicosatrienoic acid; EpODE, epoxy-octadecadienoic acid; EpOME, epoxy-octadecenoic acid; ETA, eicosatetraenoic acid; GLA, gamma-linolenic acid; HDoHE, hydroxy-docosahexaenoic acid; HDHA, hydroxy-docosahexaenoic acid; HEPE, hydroxy-eicosapentaenoic acid; HETE, hydroxy-eicosatetraenoic acid; HETrE, hydroxy-eicosatrienoic acid; HODE, hydroxy-octadecadienoic acid; HOTrE, hydroxy-octadecatrienoic acid; LA, linoleic acid; SDA, stearidonic acid; TriHOME, trihydroxy-octadecenoic acid, PG, prostaglandin; LT, leukotriene; TX, thromboxane.

PUFAs can be classified into two major families according to the position of the last double bond with respect to the methyl terminus of the acyl chain, being either n-3 or n-6 PUFAs. Both follow parallel metabolic pathways involving the same enzymes (**Figure 2**). Alpha-linolenic acid (ALA, 18:3n-3) and linoleic acid (LA, 18:2n-6) are metabolized to stearidonic acid (SDA, 18:4n-3) and gamma-linolenic acid (GLA, 18:3n-6), respectively, by the delta-6-desaturase (D6D). This represents the slowest step in the metabolic pathway, and therefore D6D activity partly regulates the PUFA biosynthetic pathway (47). Interestingly, D6D has a higher affinity for LA than for ALA (48). Moreover, the human epidermis has a low delta-5-desaturase (D5D) and D6D activity, which makes the production of long-chain n-3 PUFAs dependent on food intake (49). Both products then undergo an elongation cycle to produce eicosatetraenoic acid (ETA, 20:4n-3) and dihomo-gamma-linolenic acid (DGLA, 20:3n-6), respectively, followed by desaturation by D5D to form eicosapentaenoic acid (EPA, 20:5n-3) and arachidonic acid (AA, 20:4n-6). These metabolites are subsequently transformed into n-3 docosapentaenoic acid (n-3 DPA, 22:5n-3) and docosatetraenoic acid (DTA, 22:4n-6), and into docosahexaenoic acid (DHA, 22:6n-3) and n-6 DPA (22:5n-6). This last reaction is a β-oxidation requiring the transfer of precursors from the endoplasmic reticulum to the peroxisomes (47). Retro-conversion mechanisms can also occur, as DHA can be converted to EPA. However, these reactions are not very efficient (48). N-3 and n-6 PUFAs have structural and metabolic roles in the body: they serve as energy sources, are major constituents of cell membranes, are involved in gene regulation and have roles in cellular response regulation (50).

N-3 and n-6 PUFAs are crucial structural constituents of the skin, as they are found in phospholipids, triglycerides, ceramides and as free fatty acids (51). When present in phospholipids, PUFAs influence the physical properties of biological membranes and modulate membrane organization, ionic permeability, elasticity, and microdomain formation (52). Indeed, when the level of unsaturation of fatty acids increases, the lipid membrane organization is affected, resulting in increased phospholipid membrane fluidity. The membrane fluidity induced by PUFAs is known to interfere with various receptors, such as insulin and T-cell receptors (48). LA alone accounts for 15% of fatty acids in the epidermis, while AA, EPA, and DHA account for 5% (11). The importance of LA for skin barrier formation has been extensively investigated over the years (53). The main symptom of dietary linoleic acid deficiency in rodent is a drastic increase in skin permeability and the appearance of scaly patches (1, 54). Histologically, a deficiency in LA results in thickening of the skin (acanthosis) and a decrease in the thickness of the granular and cornified layers. Moreover, although lamellar bodies are synthesized normally, they are practically empty of content. Hence, their extrusion does not adequately fill the intercellular spaces (55). Furthermore, LA is used to produce ω-hydroxylated ceramides, essential for the scaffolding of the skin lipid matrix (36). The lack of ω-hydroxylated ceramides does not allow the organization of the lamellar bilayer, thus causing a disorganization of the stratum corneum and a decrease in its impermeability.

### Bioactive lipid mediators

A wide range of bioactive lipid mediators can be produced from the oxygenation of the free form of ALA, EPA, DHA, LA, DGLA, and AA by cyclooxygenases (COXs), lipoxygenases (LOXs), cytochrome P450 (CYP), or non-enzymatic reactions (**Figure 2**) (56). Specifically, COXs allow the generation of prostanoids, including prostaglandins (PGs), prostacyclins and thromboxanes (TXs). LOXs convert PUFAs into hydroxy-fatty acids (HFAs), leukotrienes (LTs), eoxins, lipoxins (LXs), trioxilin, hepoxilins, resolvins (Rvs), protectin (PD) and maresins (MaRs). CYP converts PUFAs to other types of HFAs and to epoxygenated FAs (EET). PUFAs can also be oxidized to an array of lipid mediators following non-enzymatic reactions, such as to isoprostanes (57, 58). Finally, other lipid mediators can also be produced from phospholipids that contain PUFAs, such as endocannabinoids, N-acylethanolamines (NAEs) and monoacylglycerols (MAGs) (59). Bioactive lipid mediators can also be classified according to the number of carbons they contain. Lipid mediators with 18 carbons (ALA- and LA-derived), 20 carbons (EPA-, DGLA-, and AA-derived) and 22 carbons (DHA-derived) are named octadecanoids, eicosanoids and docosanoids respectively (2). AA-derived eicosanoids are usually present in the greatest quantities and are therefore more studied in the literature. Indeed, recent lipidomic analyses have shown that AA-derived eicosanoids alone account for nearly half of all bioactive lipid mediators (2).

#### Prostanoids

Prostanoids are generated by COX types 1 (COX1) and 2 (COX2). COX1 is a constitutive enzyme producing basal levels of prostanoids, while COX2 is a non-constitutive enzyme, and its expression is modulated by external stimuli to regulate inflammatory responses (60). COXs convert EPA, DGLA and AA into PGH, which is subsequently transformed by the various prostaglandin synthases into PGE, PGD, PGF, PGI, TXA and TXB. EPA, DGLA and AA are respectively metabolized into series 3, series 1 and series 2 prostanoids (61). Prostanoids are characterized by their 5-carbon ring structure (2). They regulate a wide variety of mechanisms primarily through their binding to specific prostanoid receptors. These GPCRs are divided into five classes based on their affinity for PGD, PGE, PGF, PGI or thromboxanes, namely DP, EP, FP, IP, and TP receptors respectively. EP receptors are divided into four subtypes, namely EP1, EP2, EP3 and EP4. DPs are divided into two subtypes: DP1 and DP2 (62).

The broad understanding of prostanoid metabolism is attributable to the 1970s discovery that COXs are therapeutic targets of aspirin (63). Prostanoids have been widely studied ever since and it has emerged that at low concentrations, they regulate skin biological processes, whereas at high concentrations, they regulate inflammatory responses (64). AA-derived PGE_2_ is one of the major eicosanoids produced in the skin by both fibroblasts and keratinocytes, and it is one of the most studied prostanoids (65–67). PGE_2_ is involved in the regulation of the proliferation and differentiation of epidermal keratinocytes (68, 69). Moreover, PGE_2_ has vasodilatory properties, can regulate the migration and maturation of Langerhans cells and was found to be involved in skin aging (70–72). PGF_2α_ and PGD_2_ are also major PGs found in the skin (73). The series-3 prostanoids also bind to the various prostanoid receptors, but with less affinity than the series-2 prostanoids. Consequently, their individual role has been little studied since it is assumed that they exert similar functions to their n-6-derived congeners.

#### Hydroxy-fatty acids and leukotrienes

**Figure 3:**
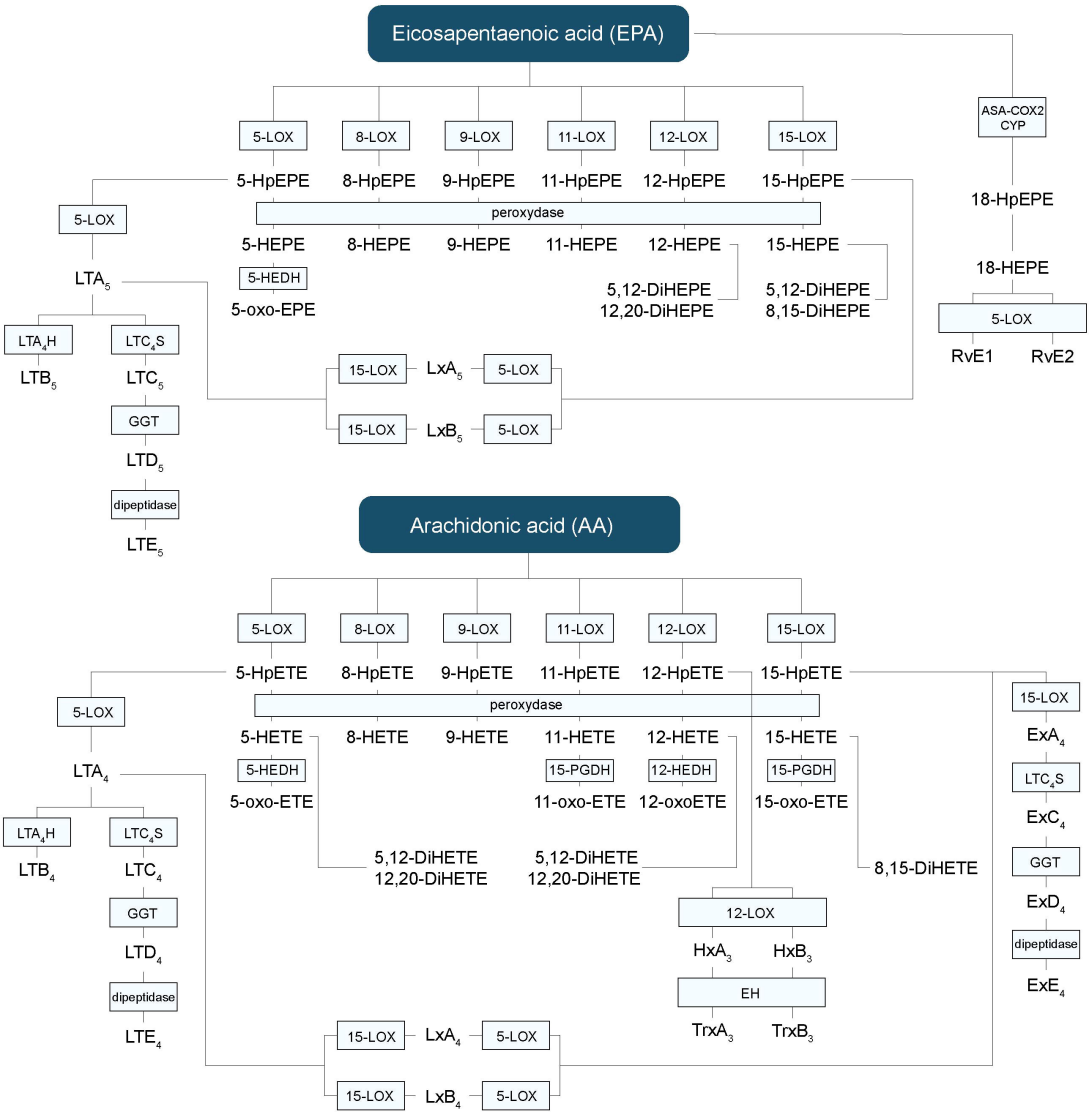
Hydroxy-fatty acids, leukotrienes and other lipid mediators’ biosynthetic pathways derived from EPA and AA. AA, arachidonic acid; CYP, P450 cytochrome; DiHETE, dihydroxyeicosatetraenoic acid; DiHEPE, dihydroxyeicosapentaenoic acid; EPA, eicosapentaenoic acid; Ex, eoxin; HEPE, hydroxy-eicosapentaenoic acid; HETE, hydroxy-eicosatetraenoic acid; HpETE, hydroxy-peroxy-eicoisatetraenoic acid; HpEPE, hydroxy-peroxy-eicoisapentaenoic acid; LT, leukotriene; LOX, lipoxygenase; Lx, lipoxin; oxo-ETE, oxo-eicosatetraenoic; oxo-HEPE, oxo-eicosapentaenoic.

**Figure 4:**
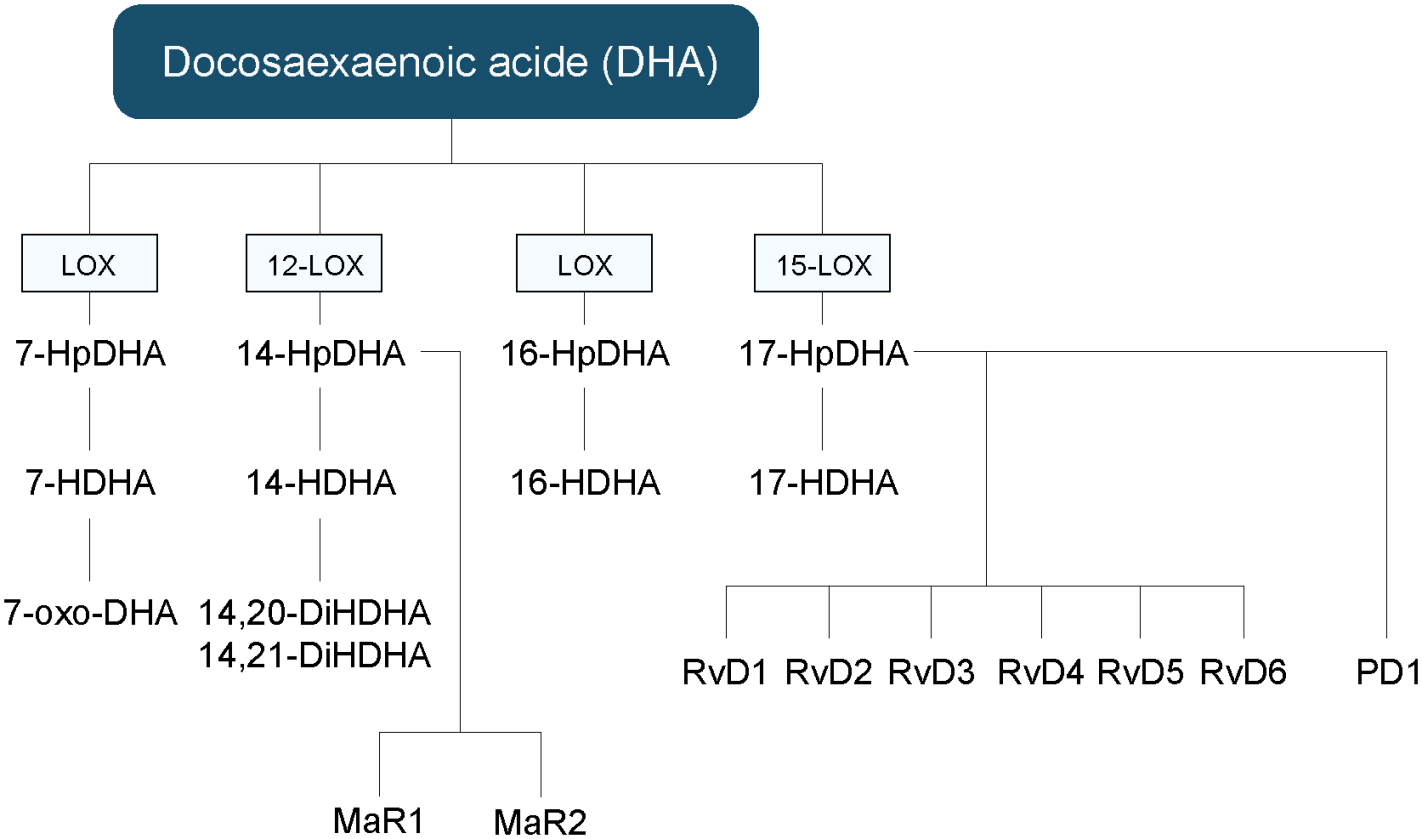
Hydroxy fatty acids, and other lipid mediators’ biosynthetic pathways derived from DHA. DHA, docosahexaenoic acid; HDHA, hydroxy-docosahexaenoic acid; HpDHA, hydroxy-peroxy-docosahexaenoic acid; MaR, maresin; oxo-DHA, oxo-octadecatrienoic; PD, protectin; Rv, resolvin.

LOXs can generate a complex and elaborate network of bioactive lipid mediators beginning with the generation of mono-, di- or tri-HFAs (**Figure 3**) (2). They are classified according to the position of oxygen insertion in the PUFA, namely 5-LOX, 8-LOX, 9-LOX, 11-LOX, 12-LOX, and 15-LOX (74). There are two isoforms of 15-LOX, 15-LOX-1 and 15-LOX-2, and two isoforms of 12-LOX. LOXs transform ALA into hydroxy-octadecatrienoic acids (HOTrEs), EPA into hydroxy-eicosapentaenoic acids (HEPEs), DHA into hydroxy-docosahexaenoic acids (HDHAs) (**Figure 4**), LA into hydroxy-octadecadienoic acids (HODEs), DGLA into hydroxy-eicosatrienoic acids (HETrEs) and AA into hydroxy-eicosatetraenoic acids (HETEs) *via* their respective unstable peroxidized forms (HpOTrE, HpEPE, HpDHA, HpODE, HpETrE, and HpETE) (75). Keto derivatives (oxo) can then be produced *via* peroxidases from the HFAs (oxo-OTrEs, oxo-EPEs, oxo-DHAs, oxo-ODEs, oxo-ETrEs, oxo-ETEs) (2). Of note, some HFAs such as AA-derived 11-HETE, DHA-derived 13-HDHA and LA-derived 9-HODE can also be synthesized by COXs. However, these biosynthetic pathways are secondary (76, 77). PUFAs can also be transformed by LOXs into LTs, hepoxilins and eoxins (2). Indeed, EPA-derived 5-HpEPE, DGLA-derived 5-HpETrE, and AA-derived 5-HpETE, can be converted by 5-LOX activating protein (FLAP) into LTs of the 5-, 6-, and 4-series respectively (78). First, hydroperoxy-FAs are transformed into LTAs, which are converted to LTBs by LTA_4_ hydrolase (LTA_4_H) or to LTC, LTD, and LTE by successive conversions of LTC_4_ synthase (LTC_4_S), gamma-glutamyltransferase, and dipeptidase (2). The LTs can be divided into two main groups, one composed of LTB_4_ only and the second one containing cystenyl LTs (Cys-LTs), i.e., LTC_4_, LTD_4_ and LTE_4_ (**Figure 3**) (79). A leukotriene-like pathway is also taken by AA-derived 15-HpETE to form eoxins A_4_, C_4_, D_4_, and E_4_. The receptors activated by HFAs remain poorly understood. They are known to act as signaling mediators by activating GPCRs and to be ligands activating peroxisome proliferator-activated receptors (PPARs) (75). LTB_4_ binds to high-affinity LTB_4_ (BLT1) and low-affinity LTB_4_ (BLT2) receptors (79). All Cys-LTs bind to CysLT1R, CysLT2R, and CysLT3R receptors (79).

HFAs and their derivatives are involved in the regulation of several biological processes, including skin homeostasis. In general, HFAs produced by 12-LOX are mostly associated with pro-inflammatory properties, whereas those derived from 15-LOXs are better known for their anti-inflammatory properties (73). In the skin, both 12- and 15-LOX are very active, and 12-LOX plays a key role in the regulation of cell survival (75, 80). 12-HETE is present in large quantities in the skin (81, 82). It is involved in the proliferation of epithelial cells and in the synthesis of dermal extracellular proteins (83, 84). Similarly, epidermal 15-LOXs produce significant levels of 15-HEPE, 13-HODE, 15-HETE and 17-HDHA (73). The production of 15-HETE by dermal cells decreases the growth of epidermal cells by reducing the expression of 12-LOX (75, 85). 13-HODE has antiproliferative properties in the epidermis (86). Of note, 12- and 15-LOX metabolism differs between humans and rodents. Indeed, leukocyte-type 12/15-LOX synthesizes both 12- and 15-HETE in rodents, unlike in humans, which might lead to different HFA profiles in mice (87). The level of activity of 5-LOX in the healthy skin is low and is mainly associated with differentiated keratinocytes, Langerhans cells, and leukocytes upon infiltration (88). Different enantiomers of HFAs may have different effects (89). 8-, 9- and 11-HETE are also found in the skin (90). Finally, some HFAs can be incorporated into the phospholipids of cell membranes, including those of keratinocytes. Indeed, relatively high amounts of 15-HETE, 5-HETE, 9-HODE, and 13-HODE are found in the *sn*-2 position of phospholipids (91, 92). LTs are known for their inflammatory properties. Indeed, the BLT1 receptor is predominantly expressed on leukocytes, and its activation by LTB_4_ is involved in the regulation of chemotaxis and the activation of these cells. Little is known about the effect of BLT2 receptor activation (79). Both receptors are also expressed in keratinocytes, but not in fibroblasts (93). Activation of the BLT2 receptor with an agonist induces the migration of keratinocytes and the stimulation of transforming growth factor-β1 (TGF-β1) production, thereby stimulating fibroblast proliferation (94). Series 5 LTs derived from n-3 PUFAs also exert inflammatory effects by activating the same receptors as those of series 4. However, their affinity for these receptors is 100-fold lower than for series 4 LTs, resulting in a lesser inflammatory effect (50).

#### Specialized pro-resolving mediators

PUFAs can be transformed by the LOXs into SPMs, including LX, Rvs, PD and MaRs (2). AA is converted to LXA_4_ and LXB_4_ (95). EPA is converted to 18-HEPE by acetylated COX-2 or CYP, as well as in RvE1-5, LXA_5_ and LXB_5_ (96, 97). DHA can be converted into PD1, MaR1, MaR2 and RvD1-6 (98, 99).The RvEs and RvDs are synthesized by the combined action of the 5-, 12- and 15-LOX. However, their exact biosynthetic pathways are still only partly defined. In the inflammatory process, RvE1 is known to bind to ChemR23 receptors to generate resolving responses, while RvD1 interacts with ALX/FPR2 and DRV1 in a context-specific manner (100, 101). RvE1 also binds to BLT1 and ERV1 receptors (96, 101, 102). RvD2 was shown to be a potent ligand for GPR18 also known as DRV2 (103). Additionally, lipoxins exert their anti-inflammatory action by binding to the ALX receptor (104). LXA_4_ can also serves as an endogenous ligand of the endocannabinoid receptor CB_1_ (102, 105).

The main SPMs that were detected in healthy human skin are the RvD5 and MaR2 (90). Levels of other SPMs, such as RvE1, RvD1 or PD1 were not detected in human skin and plasma samples even after n-3 PUFA supplementation (106). The ALX/FPR2 and DRV2 receptors were found in the epidermal layer of human skin (107).

#### Endocannabinoids, monoacylglycerols and N-acylethanolamines

The discovery of CB_1_ and CB_2_ receptors led to the identification of endogenous molecules able to bind to these proteins, namely arachidonoyl-ethanolamide (AEA) and 2-arachidonylglycerol (2-AG), both derived from AA (108, 109). 2-AG and AEA are the two classic endocannabinoids. AEA is part of the larger NAE family while 1- and 2-AGs are part of the MAG family (110). Over the past 15 years, many molecules from both families have been further characterized (111–113). Since these new mediators share similar functions with endocannabinoids, they have been grouped under the term endocannabinoidome (114, 115). NAEs can be biosynthesized through 4 pathways. The most studied pathway begins with the transfer of an acyl chain from a phospholipid to the primary amide of a phosphatidylethanolamine by calcium-dependent transacylase (CDTA) to form an *N*-acylphosphatidylethanolamine (NAPE). Then, a PLD hydrolyzes the NAPE to generate the NAE (116). MAGs are generated from a phospholipid that is hydrolyzed by phospholipase C to form a DAG, and then transformed to a MAG by DAGL. NAEs and MAGs are produced and then rapidly inactivated by fatty acid amide hydrolase (FAAH) and monoacylglycerol lipase (MAGL), respectively (116). The effect of endocannabinoids is mediated by the activation of the cannabinoid receptors, CB_1_ and CB_2_, both expressed in the skin (117). Additionally, other less specific receptors can be stimulated by endocannabinoids such as Transient Receptor Potential Vanilloid-1 (TRPV1), 5-hydroxytriptamine (5-HT), PPARγ, and PPARα (118).

Although endocannabinoids are usually related to the stimulation of the nervous system, recent evidence has shown that they may also have an important role to play in skin homeostasis (119, 120). The endocannabinoid signaling system is fully present in both keratinocytes and fibroblasts (121, 122). Both AEA and 2-AG are found in the dermis and the epidermis (56, 90, 106). The main NAEs found in the skin are the palmitoyl-ethanolamide, followed by steroyl-ethanolamide, oleoyl-ethanolamide, linoleoyl-ethanolamide, AEA, docosahexaenoyl-ethanolamide and eicosapentaenoyl-ethanolamide (123). Similarly, the predominant MAGs in the epidermis are the 2-palmitoyl-glycerol, followed by 2-oleoyl-glycerol, 2-linoleoyl-glycerol, 2-AG, 2-docosahexaenoyl-glycerol and 2-eicosapentaenoyl-glycerol (90). Recently, two new molecules, namely 13-HODE-EA and 13-HODE-G, were characterized and identify in healthy skin (124, 125). The CB_1_ receptor is located in the spinous and granular layers of the epidermis, as well as in differentiated cells of the sebaceous glands and hair follicles (117). The CB_2_ receptor, on the other hand, is expressed in the basal layer of the epidermis as well as in undifferentiated cells of the sebaceous glands and hair follicles (126). The CB_2_ receptor is also abundantly expressed in immune cells (127). TRPV1 receptors are expressed primarily in basal layer keratinocytes, but low levels of expression have also been observed in supra-basal layer keratinocytes (128). Endocannabinoids are thought to be involved in the regulation of the epidermal differentiation process (118, 129).

## Psoriasis

Psoriasis is an autoimmune skin disease affecting about 3% of the worldwide population (130). This condition is characterized by the appearance of erythematous plaques covered with whitish scales (131). The severity of the disease varies significantly between individuals, leading to several classifications. According to the National Psoriasis Foundation, psoriasis is considered mild when less than 3% of the body surface is affected, moderate when 3 to 10% is affected, and severe when it affects more than 10%. The Psoriasis Area Severity Index (PASI) is a clinical tool commonly used to assess and classify severity (132). Psoriasis is a multifactorial condition associated with various comorbidities, the most common being psoriatic arthritis, which affects 5 to 20% of psoriatic patients (131). Other associated comorbidities include cardiovascular disease, metabolic syndromes, Crohn’s disease, type 2 diabetes, obesity, cancer, and depression (133, 134). The exact mechanisms triggering psoriasis remain unknown (131), however, it is well established that a combination of environmental and genetic factors is involved. The most common environmental factors involved are skin infections, injuries or trauma, psychological stress, medications, alcohol consumption, and smoking (131, 133). No definitive effective cure for psoriasis has yet been identified. Treatment is therefore based on a wide range of therapeutic approaches, including topical treatments, phototherapy, and systemic and biological agents (135). The selection of therapy varies for each patient according to the course of the disease, the extent of the activity, the severity of symptoms, and the response to treatments. Approximately 75% of patients with psoriasis are adequately treated with topical treatments, the main ones reported being vitamin D_3_ analogues (calcitriol, calcipotriol, tacalcitol et maxacalcitol) and retinoic acids (tazarotene) (131, 136). These therapies are usually prescribed as monotherapies or with the addition of topical corticosteroids such as betamethasone, fluocinonide, and hydrocortisone, especially when using retinoids in order to reduce inflammation and irradiation (131, 132). Topical treatments, however, have limited effects when treating patients with moderate to severe psoriasis. Phototherapy or systemic drugs should be then considered (131).

The main histological features of psoriasis are hyperproliferation and the incomplete differentiation of epidermal keratinocytes, the infiltration of immune cells, and increased angiogenesis (130). Psoriatic keratinocytes in the basal and spinous layers of psoriatic skin hyperproliferate and cause epidermal hyperplasia, also described as acanthosis (137). While the epidermis of healthy skin is about 50 to 100 mm thick, the epidermis of psoriatic patients can reach about 250 mm in thickness (36, 138). The differentiation process of keratinocytes is also deregulated, resulting in the suppression of the stratum granulosum as well as the presence of undifferentiated keratinocytes in the stratum corneum (139). This abnormal differentiation of the psoriatic epidermis results in significant deregulation of the proteins of the stratum corneum. In general, proteins expressed in the early supra-basal layers of the epidermis, known as early markers of cell differentiation (involucrin and transglutaminase 1), are overexpressed, whereas proteins expressed in the later layers of the epidermis, the late markers (loricrin and filaggrin), have decreased expression (140). Dermal blood vessels are elongated in the papillary dermis region resulting in elongation of the dermal papillae (papillomatosis). In addition, the marked dilation of these blood vessels causes redness, which is noticeable in psoriatic skin lesions (130). A significant infiltration of immune cells is observed in both the dermis and the epidermis of psoriatic skin, and is mainly composed of macrophages, dendritic cells, and memory CD4+ T cells in the dermis, and of an increased quantity of CD8+ T cells in the epidermis. Neutrophils are also present in the stratum corneum (130).

### Psoriasis immunopathogenesis

**Figure 5:**
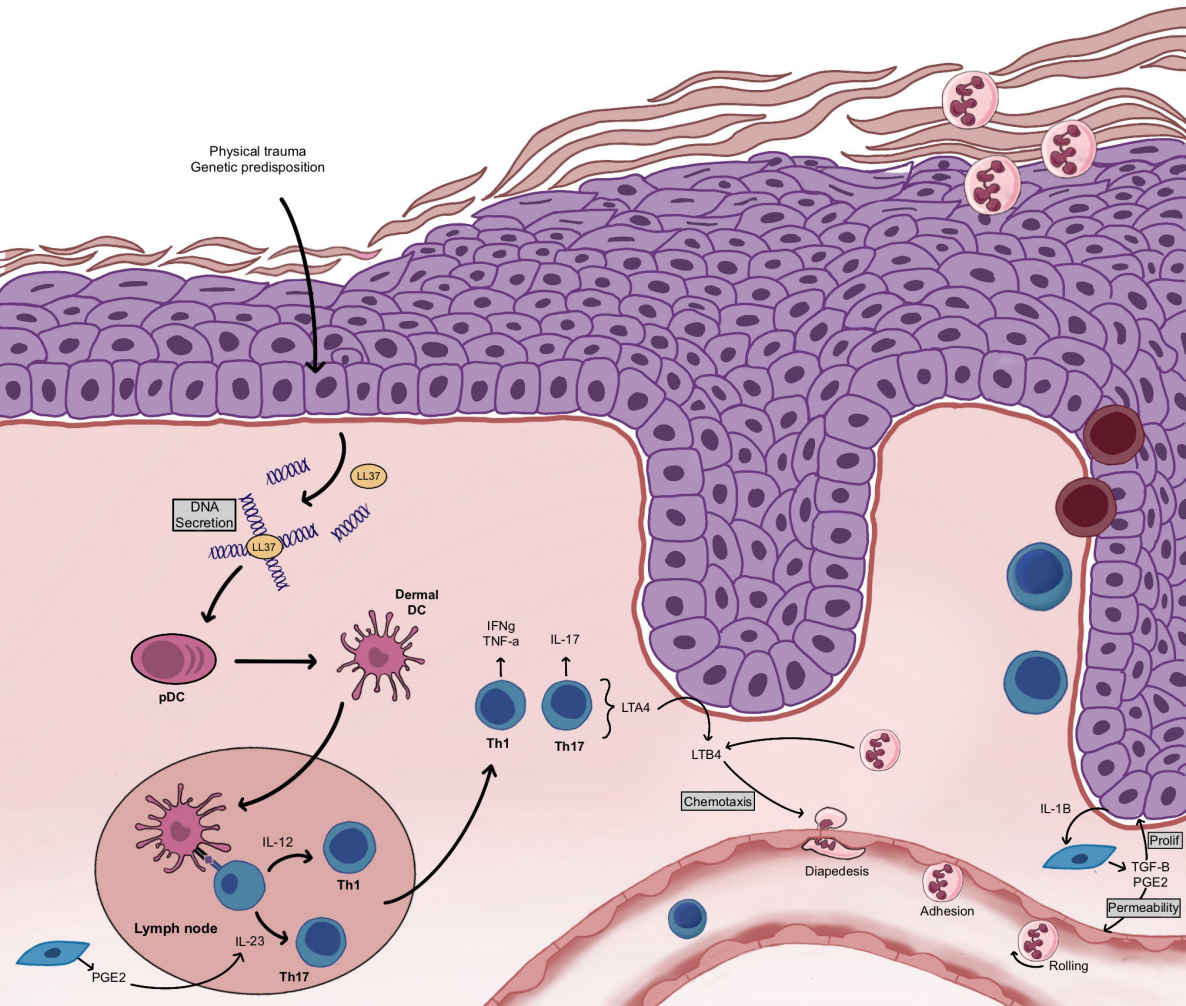
Establishment of the psoriatic plaque. Following a physical trauma or triggered by genetic predispositions, keratinocytes secrete their own DNA. The pDC become activated and stimulate the dermal DC. Dermal DC present their antigen to T cells, which induces T cell recruitment, CD4+ T cell polarization toward a Th1 and Th17 phenotype and CD8+ T cell migration to the epidermis. Keratinocytes enter into hyperproliferation. Neutrophils are recruited and migrate to the stratum corneum. LTB4 contributes to neutrophil chemotaxis while PGE_2_ contributes to keratinocyte proliferation.

Although the pathogenesis of psoriasis remains unclear, compelling experimental evidence suggests a T-cell-based immunopathogenesis (131). Nevertheless, the involvement of both epithelial and immune cells is essential for the establishment of a psoriatic lesion (141–143). The first step is the activation of cutaneous innate immunity (**Figure 5**): environmental factors induce stress, which initiates the process of lesion formation in a patient with a predisposition to psoriasis. In response, keratinocytes secrete their own deoxyribonucleic acid (DNA), which forms complexes with antimicrobial peptides (141, 144). Keratinocytes also produce cytokines, including interleukin-1β (IL-1β), IL-6, and tumor necrosis factor-α (TNF-α), and chemokines such as CCL20, CXCL9, CXCL10, and CXCL11 (31). Then, keratinocytes stimulate cutaneous dendritic cells through the secretion of various inflammatory agents. Three types of dendritic cells are found in the skin: the plasmacytoid dendritic cells, the myeloid dendritic cells of the dermis and the Langerhans cells of the epidermis. The DNA and antimicrobial peptide complexes secreted by keratinocytes activate plasmacytoid dendritic cells, which then produce interferon α (IFN-α) (145). In addition, cytokines secreted by keratinocytes and plasmacytoid dendritic cells induce the activation of myeloid dendritic cells in the dermis. These myeloid dendritic cells are antigen-presenting cells that provide the interface between innate and adaptive immunity, interacting with both CD8+ and CD4+ T cells. Then, the activation of adaptive immunity and of different types of T cells results in the formation of a complex signaling network composed of numerous cytokines and inflammatory mediators (31). The presentation of myeloid dendritic cell antigens to CD8+ T cells leads to their activation (141). The activation and expansion of CD8+ T cells causes their migration to the epidermis where they encounter major histocompatibility complex receptors present on the surface of keratinocytes, stimulating the local release of cytokines, such as IFNγ (141). These cytokines increase local inflammation and stimulate keratinocyte proliferation (31). In parallel, myeloid dendritic cells participate in the recruitment of CD4+ T cells by secreting additional pro-inflammatory mediators (IL-12, IL-23 and TNF-α), leading to the differentiation of naive CD4+ T cells into Th1 and/or Th17 T helper cells following antigen presentation (145). The differentiation of CD4+ T cells is specifically directed by cytokines secreted by the myeloid dendritic cells. In psoriasis, IL-12 and IL-23 production stimulates T cell differentiation into Th1 and Th17 cells, respectively, while few Th2 cells are observed (31). Hence, psoriasis adopts an immune profile characterized by Th1+, Th17+ and Th2- cells. Th1, Th17, and Th22 cells produce signature cytokines of their own causing inflammation amplification loops (31, 141, 146). Th1 cells produce mainly IFNγ and TNFα, while Th17 cells produce IL-17A, IL-17F and IL-22. Over the past decades, research has put forward a key role for the IL-23/IL-17 axis within psoriasis. IL-17 stimulates keratinocytes to produce TNFα and chemokines (CXCL1, CXCL3, CXCL5, and CXCL8), which stimulate the recruitment of neutrophils to the lesion and induce the increased proliferation of keratinocytes (31). Th22 cells have recently been identified in psoriatic skin. IL-22 produced by Th17 and Th22 cells contributes to the psoriatic histological phenotype, including epidermal hyperplasia, acanthosis, and parakeratosis (132, 147, 148).

### Lipid mediators dysregulated in psoriasis

The profound dysregulation of lipid metabolism in psoriatic skin has an impact on the levels of various structural lipids, such as phospholipids, triglycerides, and ceramides, and on the levels of lipids involved in inflammation, such as bioactive mediators (25, 149). Interestingly, a significant alteration of the metabolism of polyunsaturated fatty acids is found in psoriasis (**Table 1**). Indeed, a great abundance of n-6 PUFAs is measured in the blood and skin of these patients, which leads to altered levels of different types of bioactive mediators derived from n-6 PUFAs (25). AA is the n-6 PUFA that is the most dysregulated in psoriasis and many lipid mediators derived from it are found in highly increased amounts in psoriatic skin (**Table 1**) (26). These bioactive mediators play a key role as inflammatory mediators, contributing to the development of psoriatic lesions. The main AA-derived lipid mediators enhanced in psoriatic skin were reported to be the HFAs and their derivatives (167). Indeed, levels of 12-HETE were found to be increased in psoriatic skin (26, 167). 12-HETE is an important leukocyte chemoattractant and was found to promote T cell recruitment in psoriasis (168). However, finding increased amounts of 12-HETE in cutaneous wound healing prompted researchers to question whether 12-HETE production could also act as a defensive mechanism in psoriatic skin (169). Additionally, a strong increase in the quantities of LTB_4_ were reported in the skin of patients with psoriasis. This lipid mediator would mainly be produced by infiltrating neutrophils, but T cells also have significant 5-LOX activity and keratinocytes considerable LTA_4_H activity (170, 171). LTB_4_ is present in both early and chronic plaques, supporting a role in the initiation of the disease (162). The signaling pathway of LTB_4_-BLT1 in neutrophils significantly accelerates the infiltration of neutrophils in the skin in collaboration with CXCR2, a chemokine receptor for CXCL1 and CXCL2, thus leading to psoriasis (172). LTB_4_ also acts on dendritic cells and T cells through the BLT1 receptor and facilitates the migration and production of cytokines, thus contributing to the progression of psoriasis (173). Moreover, the topical application of LTB_4_ to normal skin was found to induce microabscesses, and the hyperproliferation of epidermal cells (174, 175). Lower levels of 15-HETE were found in psoriatic dermis as compared with healthy dermis (176). Given the important role of 15-HETE in the regulation of 5- and 12-LOX epidermal activity, reduced levels of 15-HETE were postulated as being involved in the enhanced 5-LOX and 12-LOX product levels found in psoriasis (85). The levels of 9-HODE and 13-HODE were also found to be increased in psoriatic skin as compared with healthy skin (25, 177). It is important to mention that great differences were reported regarding the levels of certain lipid mediators depending on the source of the biological sample, i.e., whether it came from blood, serum, plasma or skin. Therefore, the study of these differences could lead to a better understanding of lipid dysregulation in psoriasis.

**Table 1:** Summary of lipid mediators involved in psoriasis.

Markers	Biosynthesis	Receptors	Levels in psoriatic patients		Known mechanisms	References
			Blood	Skin		
***Linoleic acid-derived lipid mediators***						
9-HODE	12-LOX	TRPV1	Increased to double.	Increased compared with healthy skin.	- Facilitates the release of inflammatory cytokines	(25, 150)
13-HODE	15-LOX	PPARs	Increased to double.	Increased compared with healthy skin.	ND	(25, 150, 151)
***α-linolenic acid-derived lipid mediators***						
9-HOTrE	12-LOX	ND	ND	Tends to increase in psoriasis compared with healthy skin.	ND	(25)
13-HOTrE	15-LOX	ND	ND	Tends to increase in psoriasis compared with healthy skin.	ND	(25)
***Dihomo-γ-linolenic acid-derived lipid mediators***						
PGE_1_	COX/PGES	EP1	Increased	Increased compared with healthy skin.	- Increases cAMP levels	(151, 152)
***Arachidonic acid-derived lipid mediators***						
PGE_2_	COX/PGES	EP2-EP4	No effect	Increased in the epidermis	- Increases the production of IL-23 by dendritic cells - Increases the expression of the IL-23 receptor gene on Th17 cells - Supports the expansion of Th17 cells - Stimulate keratinocyte proliferation	(25, 26, 153)
PGF_2a_	COX/PGFS	FP	ND	Increased in psoriatic epidermis	ND	(26, 154)
8-iso-PGF_2α_	Oxidative stress	ND	ND	Increased	-Activation of TNFα	(151)
15-d-PGJ_2_	CRTH2 and PPARγ	ND	ND	Increased compared with healthy skin.	- Chemotaxis of Th2 cells, eosinophils, and basophils - Regulation of PPARγ (particularly in macrophages) -Up-regulation of NF-κB and ERK signaling pathways	(151, 155)
TXB_2_	TXs	TP	ND	Decreased compared with non-lesional skin	ND	(25, 151)
5-HETE	5-LOX	OXER1, BLT2	No effect	Slightly increased compared with healthy skin	- Activation of OXER1 receptor, resulting in cell-activation pathways such as MAPK, ERK, p38 and protein kinase B - Accumulation of monocyte-derived macrophage	(25)
8-HETE	8-LOX	PPARα	ND	Increased compared with healthy skin.	ND	(25)
9-HETE	9-LOX	RXRγ	Slightly increased	No effect	- Chemoattractant of neutrophils	(25)
11-HETE	COX2	ND	Slightly increased	Slightly decreased compared with healthy skin	11R-HETE, generated by COX-2 in epithelial cells, is also a substrate for 15-PGDH, being converted to 11-oxo-ETE.	(25, 156)
12-HETE	12-LOX	BLT2	ND	Increased compared with healthy skin.	- PMN chemoattractant	(25, 26, 150, 157, 158)
15-HETE	15-LOX	BLT2	ND	Increased compared with healthy skin.	- Anti-apoptotic activities	(159)
4-HNE	ND	ND	Increased	Increased	- Activation of the MAPK pathway	(151)
LTB_4_	5-LOX	BLT1 > BLT2	No effect	Increased compared with healthy skin.	- Most potent neutrophil chemoattractant -Facilitates the migration of dendritic cells and T cells, as well as their production of cytokines	(25, 151, 153, 157, 160)
LTC_4_	5-LOX	CysLT2>CysLT1	ND	Increased compared with healthy skin.	- Most potent eosinophil chemoattractant	(157, 160, 161)
LTD_4_	5-LOX	CysLT1	ND	Increased compared with healthy skin.	ND	(160–162)
***Eicosapentaenoic acid-derived lipid mediators***						
5-HEPE	5-LOX	GPR119- GPR120	Slightly increased	No effect	ND	(154)
12-HEPE	12-LOX	GsPCR (not identified yet)	Increased	No effect	- Downregulation of CXCL1 and CXCL2 gene in keratinocytes	(154, 163, 164)
15-HEPE	15-LOX	PPARγ	Increased	Slightly increased	- Diminution of the migration of peripheral mononuclear cells	(51, 154, 165)
18-HEPE	CYP450	ND	Slightly increased	No effect	- Repression of CXCR4 expression on lung melanoma cells - Diminution of macrophage activation	(154, 166)
***Docosahexaenoic acid-derived lipid mediators***						
4-HDHA	5-LOX	PPARγ	No difference	No difference	ND	(25, 159)
14-HDHA	12-LOX	ND	Slightly increased.	Decreased compared with healthy skin.	ND	(25)
17-HDHA	15-LOX	DRV1/DRV2	ND	Increased compared with healthy skin.	ND	(25)

COX, cyclooxygenase; GPR, G protein-coupled receptor; HEPE, hydroxyeicosapentaenoic; HETE, hydroxyeicosatetraenoic; HDHA, hydroxy-docosahexaenoic acid; HNE, hydroxyonenal; HODE, hydroxy-octadecadienoic acid; HOTrE, hydroxy-octadecatrienoic acid; IL, interleukin; LOX, lipoxygenase; LT, leukotriene; ND, not documented; PPAR, Peroxisome proliferator-activated receptor; PGJ, prostaglandin J; PGE, prostaglandin E; RXR, retinoid X receptor; TRPV1, transient receptor potential vanilloid 1; TX, thromboxane.

On the other hand, reports of prostanoid levels in psoriasis have been conflicting (25, 152, 167). Indeed, many research teams reported no alteration in PGE_2_ levels in psoriatic skin as compared with healthy skin (25, 152, 167, 178), while some teams found an increase in PGE_2_ in the psoriatic skin (26). A decreased capacity to biosynthesized prostaglandins was measured in involved and uninvolved psoriatic epidermis (152). *In vitro*, the production of PGE_2_ by fibroblasts leads to the production of IL-1, stimulating the proliferation of keratinocytes (179, 180). Moreover, the production of PGE_2_ by fibroblasts promotes the production of IL-23 by dendritic cells, which supports the expansion of Th17 cells (181, 182). Thereafter, the production of PGE_2_ by Th17 cells increases the expression of a sub-unit of the IL-23 receptor gene on Th17 cells *via* EP2 and EP4 receptors, facilitating the generation of Th17 cells *in vitro* (181, 182). More recently, the levels of lipid mediators derived from n-3 PUFAs were also documented as being altered in psoriatic skin (25). Among these, the levels of 7-, 14- and 17-HDHA were found to be increased in psoriatic skin as compared with healthy skin, with 14-HDHA having the most striking changes (25).

Finally, large amounts of reactive oxygen species (ROS) found in psoriasis can affect membrane phospholipids, leading to lipid peroxidation and the generation of reactive aldehydes, such as 4-hydroxynonenal (4-HNE). In fact, it was reported that 4-HNE and 8-isoPGF_2α_ are up-regulated in psoriatic skin (151). Although the ratio of n-3 to n-6 PUFAs is already known to be greatly diminished in psoriatic skin, the amounts of n-3 PUFAs as well as their role in the establishment of psoriatic lesions has been poorly studied.

The lipid dysregulation observed in psoriasis could be the result of the altered expression of proteins involved in the biosynthesis of lipid mediators, as well as the inflammatory milieu engendered by psoriatic epithelial cells. Interestingly, in various studies genes coding for different phospholipases have been listed as twenty of the most highly dysregulated genes in psoriasis (183). Protein analyses showed an increased number of different types of PLA_2_ in psoriatic skin, according with the increase of the different lipid mediators measured in psoriatic skin (151). Other genes that are dysregulated in psoriatic skin include ALOX12B, which may be responsible for the increased amount of 12-HETE (78). *In vitro*, transcriptomic analyses comparing the genetic expression profile of healthy skin with that of the psoriatic substitutes produced by tissue engineering demonstrated the dysregulation of genes involved in lipid metabolism, particularly in the PUFAs of psoriatic patients (184). Most of the dysregulated genes in psoriasis have been reviewed elsewhere (185).

### Effects of PUFA and bioactive lipid mediator interventions in psoriasis

n-3 PUFAs are known for their anti-inflammatory potential. However, their effectiveness is quite controversial despite the growing body of scientific data showing their beneficial effects. Clinical studies have investigated the effects of n-3 PUFAs, mainly EPA and DHA, on symptoms in psoriatic patients (**Table 2**). Several studies have shown that oral treatment with n-3 PUFAs improves the PASI score, erythema, scaling, itching, the extent of affected surfaces, and the amount of immune cell infiltration (187, 202, 203), whereas other studies have found no significant effects (191, 204).

**Table 2:** Summary of studies examining the effect of PUFA interventions in psoriasis.

PUFAs or lipid mediators	Clinical trials or preclinical study models	Treatments	Effects	References
***Human clinical trials***				
ALA	Human clinical trials	2600 mg ALA (12 weeks)	Significant clinical improvement of the PASI	(186)
EPA	Human clinical trials	180 mg (12 weeks)	↓ itching after 12 weeks.	(187)
15-HETE	Human clinical trials	Subcutaneous injection of 1 mmol/L of 15-HETE	↓ extent of plaques of two out of 13 patients	(157)
EPA-DHA	Human clinical trials	1200-1800 mg (8 weeks)	↓ erythema and skin scaling	(188)
		2400-3600 mg (15 weeks)	↓ body surface area of psoriasis	(15)
		15% EPA and 10% DHA (4 weeks)	↓ erythema and desquamation in patients	(189)
		2100-21000 mg (2 weeks)	Moderate improvement in clinical manifestations ↓ disease severity	(190)
		320-510 mg (16 weeks)	↓ scaling and redness ↓ cellular infiltration.	(191)
		132-240 mg (36 weeks)	No significant effects	(192)
		80-650 mg (4 weeks)	↓ scalp lesion, target plaque erythema lesion, infiltration, and scaling	(193)
		1800 mg (12 weeks)	↓ PASI, erythema, duration, scaling, and extent of area involved	(194)
***Animal models***				
RvE1	Imiquimod-induced psoriasis mice model	Intravenous administration of 200 ng per mouse 30 min before IMQ application	↓ epidermal hyperplasia ↓ IL-23 mRNA expression ↓ IL-23 production by dendritic cells *in vitro* ↓ migration of cutaneous DCs and γδ T cells	(173)
MaR1	Imiquimod-induced psoriasis mice model	Topical application of 100 ng per ear 30 min prior IMQ application each day for 5 consecutive days	↓ ear swelling response ↓ IL-17A production by γδTCR^mid+^ and CD4+ cells ↓ IL-23 receptor by suppressing RORγt	(195)
PD1	Imiquimod-induced psoriasis mice model	0.01-1 μg/kg injected subcutaneously for 7 consecutive days	↓ skin thickness, redness and scaling ↓ IL-1β, IL-6, IL-17 and CXCL1 mRNA expression ↓ STAT1 and NF-κB signaling pathway ↓ CD4^+^IFN-g^+^IL-17^+^ T lymphocytes	(196)
RvD1	Imiquimod-induced psoriasis mice model	Intraperitoneal administration of 1-5 μg/kg 1h prior to IMQ application	↓ IMG-induced psoriasiform dermatitis ↓ IMG-induced activation of ERK1/2, p38, JNK and NF-κB	(197)
RvD3	Imiquimod-induced psoriasis mice model	Single and repeated systemic administration. 2.8 mg/kg	↓ TRPV1-dependent acute pain and itch in mice	(198)
***In vitro models***				
ALA	Human reconstructed psoriatic skin model	10 μM	↓ keratinocyte proliferation ↑ keratinocyte differentiation (increased late differentiation markers such as filaggrin) ↑ ERK1/2 phosphorylation	(199)
	Human reconstructed psoriatic skin model with T cells	10 μM	↓ keratinocyte proliferation ↑ keratinocyte differentiation ↓ T cell migration in the epidermis ↓ inflammatory cytokines and chemokines (CXCL1, IL-6, IL-8)	(200)
DHA	Human reconstructed psoriatic skin model	10 μM	↓ keratinocyte proliferation ↑ keratinocyte differentiation ↓ TNF-α, COX2 and PPARδ ↑ PPARγ	(201)

↑ Increased; ↓ Decreased. ALA, alpha-linolenic acid; DHA, docosahexaenoic acid; EPA, eicosapentaenoic acid; HETE, hydroxyeicosatetraenoic; MaR, maresin; NSAIDs, non-steroidal anti-inflammatory drugs; PASI, psoriasis area and severity index; PD1, protectin D1; Rv, resolvin.

*In vitro* and *in vivo* studies have investigated the mechanisms of action underlying the beneficial effects of n-3 PUFAs in psoriasis (**Table 2**). The effectiveness of n-3 PUFAs on psoriasis was shown using a tissue-engineered psoriatic reconstructed skin model (205). The anti-psoriatic potential of ALA was investigated, showing that ALA reduces psoriatic keratinocyte proliferation by decreasing the production of pro-inflammatory lipid mediators (199). Similarly, DHA also alleviated the psoriatic features of the reconstructed psoriatic skin model, mainly by decreasing proliferation and improving the differentiation of psoriatic keratinocytes (201). Psoriasis is characterized by a strong increase in n-6-derived pro-inflammatory lipid mediators in the blood and lesional skin of patients (25). Therefore, in many studies the primary mechanism of action of an n-3 PUFA treatment is considered to be a decrease in these pro-inflammatory mediators through competition for enzymes in their metabolic pathway (199, 206). However, there is a lack of consensus among the various studies found in the literature. Additionally, n-3 PUFAs seem to affect the immune system (34). Indeed, ALA was showed to affect T cell functions in the reconstructed psoriatic skin model, mainly by decreasing the production of inflammatory cytokines (200). The impact of n-3 PUFAs on the adaptive immunity have also been studied in other models. Studies in humans have shown that dietary supplementation with fish oils decreases the relative percentage of CD4+ cells in peripheral blood, resulting in a decreased immune response (207). Moreover, a diet rich in n-3 PUFAs can reduce the expression of the class II histocompatibility major complex (CMH-II) on dendritic cells and other antigen-presenting cells, thus diminishing the likelihood of stimulating CD4+ cells (208). These studies also showed that the pre-treatment of endothelial cells with DHA decreases the functional adherence of T cells to endothelial cells *in vitro* (208, 209). Additionally, a high level of consumption of n-3 PUFAs was reported to reduce the production of TNF-α, IL-4, IL-6 and IL-1β (207, 210). Otherwise, n-3 PUFAs can activate nuclear receptors, such as the PPARs, which intervene in the regulation of the transcription of many genes involved in the metabolism of lipids (207). EPA and DHA are also recognized as interrupting the autocrine IL-2 pathway (211). PPARγ may inhibit the nuclear factor of activated T cells and could thus decrease the production of IL-2 (212). Finally, the polarization of CD4+ T cells into either Th17 or regulatory T cells (Treg) is partly governed by PPARγ activity (213). Honda et al. suggested that PGE_2_ produced by fibroblasts promotes the development of psoriatic dermatitis through the regulation of the IL-23 and IL-17 pathways. Therefore, a diet rich in n-3 PUFAs could directly alter those pathways by diminishing the production of PGE_2_ (153). In IMQ-induced psoriatic FAT-1 mice, increased n-3 PUFA production was associated with a significantly reduced number of Th17 cells, and an increased number of Tregs in the spleen (214). Of note, FAT-1 mice are genetically modified mice capable of producing n-3 PUFAs from n-6 PUFAs since they have been engineered to express the *C.elegans* n-3 fatty acid desaturase gene (fat-1), thus increasing the levels of n-3 PUFAs without the need for oral supplementation (214). However, PGE_2_ seems to be a limited therapeutic target in the treatment of psoriasis. Indeed, non-steroidal anti-inflammatory drugs such as aspirin already target prostaglandin biosynthesis, *via* cyclooxygenase inhibition. Unfortunately, these drugs does not resolve immune-mediated disorders on their own, making them suboptimal for psoriasis treatment (215). Recent studies have also investigated the specific effects of bioactive lipid mediators (**Table 2**). Treatment by subcutaneous injection of 1 mmol/L of 15-HETE was shown to be effective, reducing the extent of plaques of two out of 13 patients with psoriasis (157). In 2018, Sawada et al. reported that the administration of RvE1 in an imiquimod-induced mouse psoriatic model inhibits the production of IL-23 by dendritic cells and accordingly decreases psoriatic features (173). It has also been shown that RvD3 reduces acute pain and itch in mice in the context of psoriasis (198). Additionally, the administration of RvE1 strongly inhibits the expression of IL-23 and IL-17 in psoriatic skin (216). Finally, phototherapy, including ultraviolet B (UVB) and psoralen ultraviolet A (PUVA), has been widely used to treat psoriatic lesions (217). Interestingly, the mechanism underlying phototherapy may involve lipid mediator signaling. Indeed, UVB, UVA and PUVA all decreased 12-LOX expression and increased 15-LOX expression in keratinocytes (158).

Interestingly, n-3 PUFAs also seem to possess therapeutic potential in other pathologies and comorbidities related to psoriasis, including atherosclerosis (218). In fact, the positive effects of EPA on inflammatory diseases have been put forward in the Reduction of Cardiovascular Events with Isopent Ethyl-Intervention Trial (REDUCE-IT). This study showed that the daily consumption of 2 g of EPA ethyl ester by patients with established atherosclerosis heart disease was associated with an absolute 4.8% reduction in cardiovascular events (8). Similar to statins, n-3 PUFAs were shown to have pleiotropic effects and to attenuate the atherogenic response (219). Moreover, hypertriglyceridemia, which is characterized by a severe elevation of triglyceride levels and is associated with obesity, can be partially reduced with the consumption of 4 g/day of EPA and DHA (220). It is also important to mention that the n-3 PUFA index, which measures the percentage of long-chain n-3 PUFAs EPA and DHA in red blood cell membranes, greatly influences an individual’s response to dietary n-3 PUFA supplementation. Indeed, individuals with lower n-3 PUFA baseline before the oral supplementation tend to respond better to the n-3 PUFA supplementation than those with a naturally high baseline (221, 222).

## Conclusion

In conclusion, the roles of n-3 and n-6 PUFAs in the skin are numerous and complex. This study summarizes current findings on the implication of n-6 and n-3 PUFA lipid mediators in the skin and more precisely, their involvement in psoriasis. Findings on new bioactive lipid mediators are gradually being reported, however the underlying mechanisms have yet to be revealed. The dietary intake of n-3 and n-6 PUFAs greatly influences skin homeostasis, both in healthy and psoriatic skin. Recent advances have shown the potential of n-3 PUFAs as a treatment for psoriatic patients, notably in the reduction of the production of pro-inflammatory cytokines and the alteration of T cell functions, as well as in the reduction of the formation of inflammatory bioactive lipid mediators. Thus, deepening knowledge on the role of n-3 PUFAs in psoriasis may lead to the discovery of novel therapeutic targets for the disease.

## Author contributions

Conceptualization, MS, SM, ZR, and RP. Visualization, MS. Formal analysis, MS. Data curation, MS, SM, ZR, and RP. Writing—original draft preparation, MS, SM, and ZR. Writing—review and editing, MS, SM, ZR, and RP. Supervision, RP. All authors have read and agreed to the published version of the manuscript.

## Funding

This work was supported by the Canadian Institutes of Health Research (CIHR) grant number MOP-311262 to RP. MS and SM received studentships from the Fonds de recherche du Québec-Santé (FRQS). RP is a career award scholar from the FRQS.

## Conflict of interest

The authors declare that the research was conducted in the absence of any commercial or financial relationships that could be construed as a potential conflict of interest.

## Publisher’s note

All claims expressed in this article are solely those of the authors and do not necessarily represent those of their affiliated organizations, or those of the publisher, the editors and the reviewers. Any product that may be evaluated in this article, or claim that may be made by its manufacturer, is not guaranteed or endorsed by the publisher.

## References

[1] Burr GO Burr MM. Nutrition classics from the journal of biological chemistry 82:345-67, 1929. a new deficiency disease produced by the rigid exclusion of fat from the diet. Nutr Rev (1973) 31(8):248–9. doi: 10.1111/j.1753-4887.1973.tb06008.x 4586201

[2] Gabbs M Leng S Devassy JG Monirujjaman M Aukema HM. Advances in our understanding of oxylipins derived from dietary pufas. Adv Nutr (2015) 6(5):513–40. doi: 10.3945/an.114.007732 PMC4561827 26374175

[3] Caligiuri SP Love K Winter T Gauthier J Taylor CG Blydt-Hansen T. Dietary linoleic acid and alpha-linolenic acid differentially affect renal oxylipins and phospholipid fatty acids in diet-induced obese rats. J Nutr (2013) 143(9):1421–31. doi: 10.3945/jn.113.177360 23902961

[4] Guillou H Zadravec D Martin PG Jacobsson A. The key roles of elongases and desaturases in mammalian fatty acid metabolism: insights from transgenic mice. Prog Lipid Res (2010) 49(2):186–99. doi: 10.1016/j.plipres.2009.12.002 20018209

[5] Bang HO Dyerberg J Sinclair HM. The composition of the eskimo food in north western greenland. Am J Clin Nutr (1980) 33(12):2657–61. doi: 10.1093/ajcn/33.12.2657 7435433

[6] Hammer A Moertl D Schlager O Matschuck M Seidinger D Koppensteiner R. Effects of n-3 pufa on endothelial function in patients with peripheral arterial disease: a randomised, placebo-controlled, double-blind trial. Br J Nutr (2019) 122(6):698–706. doi: 10.1017/S0007114519001582 31262371

[7] Van Name MA Savoye M Chick JM Galuppo BT Feldstein AE Pierpont B. A low omega-6 to omega-3 pufa ratio (n-6:n-3 pufa) diet to treat fatty liver disease in obese youth. J Nutr (2020) 150(9):2314–21. doi: 10.1093/jn/nxaa183 PMC7467848 32652034

[8] Bhatt DL Steg PG Miller M Brinton EA Jacobson TA Ketchum SB. Cardiovascular risk reduction with icosapent ethyl for hypertriglyceridemia. N Engl J Med (2019) 380(1):11–22. doi: 10.1056/NEJMoa1812792 30415628

[9] Nadeau M Fruteau de Laclos B Picard S Braquet P Corey EJ Borgeat P. Studies on leukotriene b4 omega-oxidation in human leukocytes. Can J Biochem Cell Biol (1984) 62(12):1321–6. doi: 10.1139/o84-168 6099214

[10] Maclouf J Fruteau de Laclos B Borgeat P. Effects of 12-hydroxy- and 12-hydroperoxy-5,8,10,14-eicosatetraenoic acids on the synthesis of 5-hydroxy-6,8,11,14-eicosatetraenoic acid and leukotriene b4 in human blood leukocytes. Adv Prostaglandin Thromboxane Leukot Res (1983) 11:159–62. 6221520

[11] Nicolaou A. Eicosanoids in skin inflammation. Prostaglandins leukotrienes essential Fatty Acids (2013) 88(1):131–8. doi: 10.1016/j.plefa.2012.03.009 22521864

[12] Moore SA Yoder E Spector AA. Role of the blood-brain barrier in the formation of long-chain omega-3 and omega-6 fatty acids from essential fatty acid precursors. J Neurochem (1990) 55(2):391–402. doi: 10.1111/j.1471-4159.1990.tb04150.x 2115069

[13] Balvers MG Verhoeckx KC Bijlsma S Rubingh CM Meijerink J Wortelboer HM. Fish oil and inflammatory status alter the n-3 to n-6 balance of the endocannabinoid and oxylipin metabolomes in mouse plasma and tissues. Metabolomics (2012) 8(6):1130–47. doi: 10.1007/s11306-012-0421-9 PMC3483099 23136559

[14] Blasbalg TL Hibbeln JR Ramsden CE Majchrzak SF Rawlings RR. Changes in consumption of omega-3 and omega-6 fatty acids in the united states during the 20th century. Am J Clin Nutr (2011) 93(5):950–62. doi: 10.3945/ajcn.110.006643 PMC3076650 21367944

[15] Gupta AK Ellis CN Tellner DC Anderson TF Voorhees JJ. Double-blind, placebo-controlled study to evaluate the efficacy of fish oil and low-dose uvb in the treatment of psoriasis. Br J Dermatol (1989) 120(6):801–7. doi: 10.1111/j.1365-2133.1989.tb01378.x 2667615

[16] Serhan CN Savill J. Resolution of inflammation: the beginning programs the end. Nat Immunol (2005) 6(12):1191–7. doi: 10.1038/ni1276 16369558

[17] Jozsef L Zouki C Petasis NA Serhan CN Filep JG. Lipoxin a4 and aspirin-triggered 15-epi-lipoxin a4 inhibit peroxynitrite formation, nf-kappa b and ap-1 activation, and il-8 gene expression in human leukocytes. Proc Natl Acad Sci United States America (2002) 99(20):13266–71. doi: 10.1073/pnas.202296999 PMC130622 12235371

[18] Schwab JM Chiang N Arita M Serhan CN. Resolvin e1 and protectin d1 activate inflammation-resolution programmes. Nature (2007) 447(7146):869–74. doi: 10.1038/nature05877 PMC2757086 17568749

[19] Chiurchiu V Leuti A Dalli J Jacobsson A Battistini L Maccarrone M. Proresolving lipid mediators resolvin d1, resolvin d2, and maresin 1 are critical in modulating t cell responses. Sci Transl Med (2016) 8(353):353ra111. doi: 10.1126/scitranslmed.aaf7483 PMC5149396 27559094

[20] Norris PC Skulas-Ray AC Riley I Richter CK Kris-Etherton PM Jensen GL. Identification of specialized pro-resolving mediator clusters from healthy adults after intravenous low-dose endotoxin and omega-3 supplementation: a methodological validation. Sci Rep (2018) 8(1):18050. doi: 10.1038/s41598-018-36679-4 30575798 PMC6303400

[21] Markworth JF Sugg KB Sarver DC Maddipati KR Brooks SV. Local shifts in inflammatory and resolving lipid mediators in response to tendon overuse. FASEB J (2021) 35(6):e21655. doi: 10.1096/fj.202100078R 34042218 PMC9527947

[22] Serhan CN Chiang N Van Dyke TE. Resolving inflammation: dual anti-inflammatory and pro-resolution lipid mediators. Nat Rev Immunol (2008) 8(5):349–61. doi: 10.1038/nri2294 PMC2744593 18437155

[23] Chiang N Serhan CN. Specialized pro-resolving mediator network: an update on production and actions. Essays Biochem (2020) 64(3):443–62. doi: 10.1042/EBC20200018 PMC7682745 32885825

[24] Colas RA Shinohara M Dalli J Chiang N Serhan CN. Identification and signature profiles for pro-resolving and inflammatory lipid mediators in human tissue. Am J Physiol Cell Physiol (2014) 307(1):C39–54. doi: 10.1152/ajpcell.00024.2014 PMC4080182 24696140

[25] Sorokin AV Domenichiello AF Dey AK Yuan ZX Goyal A Rose SM. Bioactive lipid mediator profiles in human psoriasis skin and blood. J Invest Dermatol (2018) 138(7):1518–28. doi: 10.1016/j.jid.2018.02.003 PMC6121727 29454560

[26] Hammarstrom S Hamberg M Samuelsson B Duell EA Stawiski M Voorhees JJ. Increased concentrations of nonesterified arachidonic acid, 12l-hydroxy-5,8,10,14-eicosatetraenoic acid, prostaglandin e2, and prostaglandin f2alpha in epidermis of psoriasis. Proc Natl Acad Sci United States America (1975) 72(12):5130–4. doi: 10.1073/pnas.72.12.5130 PMC388890 1061097

[27] Baroni A Buommino E De Gregorio V Ruocco E Ruocco V Wolf R. Structure and function of the epidermis related to barrier properties. Clin Dermatol (2012) 30(3):257–62. doi: 10.1016/j.clindermatol.2011.08.007 22507037

[28] Mélissopoulos A Levacher C. La peau: Structure et physiologie. Paris: Éditions Tec & doc (2012). 272 p. p.

[29] Ziboh VA Chapkin RS. Metabolism and function of skin lipids. Prog Lipid Res (1988) 27(2):81–105. doi: 10.1016/0163-7827(88)90006-9 3060882

[30] De Kock J Rodrigues RM Branson S Verhoye L Colemonts-Vroninks H Rombaut M. Inflammation alters the secretome and immunomodulatory properties of human skin-derived precursor cells. Cells (2020) 9(4):914. doi: 10.3390/cells9040914 PMC7226778 32276503

[31] Nestle FO Di Meglio P Qin JZ Nickoloff BJ. Skin immune sentinels in health and disease. Nat Rev Immunol (2009) 9(10):679–91. doi: 10.1038/nri2622 PMC2947825 19763149

[32] Nikzad R Angelo LS Aviles-Padilla K Le DT Singh VK Bimler L. Human natural killer cells mediate adaptive immunity to viral antigens. Sci Immunol (2019) 4(35):eaat8116. doi: 10.1126/sciimmunol.aat8116 31076527 PMC6636344

[33] Banchereau J Steinman RM. Dendritic cells and the control of immunity. Nature (1998) 392(6673):245–52. doi: 10.1038/32588 9521319

[34] Ho AW Kupper TS. T Cells and the skin: from protective immunity to inflammatory skin disorders. Nat Rev Immunol (2019) 19(8):490–502. doi: 10.1038/s41577-019-0162-3 30992525

[35] Madison KC. Barrier function of the skin: “la raison d’etre” of the epidermis. J Invest Dermatol (2003) 121(2):231–41. doi: 10.1046/j.1523-1747.2003.12359.x 12880413

[36] Bouwstra JA Honeywell-Nguyen PL Gooris GS Ponec M. Structure of the skin barrier and its modulation by vesicular formulations. Prog Lipid Res (2003) 42(1):1–36. doi: 10.1016/s0163-7827(02)00028-0 12467638

[37] Oguri M Gooris GS Bito K Bouwstra JA. The effect of the chain length distribution of free fatty acids on the mixing properties of stratum corneum model membranes. Biochim Biophys Acta (2014) 1838(7):1851–61. doi: 10.1016/j.bbamem.2014.02.009 24565794

[38] Chan A Godoy-Gijon E Nuno-Gonzalez A Crumrine D Hupe M Choi EH. Cellular basis of secondary infections and impaired desquamation in certain inherited ichthyoses. JAMA Dermatol (2015) 151(3):285–92. doi: 10.1001/jamadermatol.2014.3369 PMC4498571 25565224

[39] Uchida Y Holleran WM. Omega-o-acylceramide, a lipid essential for mammalian survival. J Dermatol Sci (2008) 51(2):77–87. doi: 10.1016/j.jdermsci.2008.01.002 18329855

[40] Elias PM Feingold KR. Lipid-related barriers and gradients in the epidermis. Ann N Y Acad Sci (1988) 548:4–13. doi: 10.1111/j.1749-6632.1988.tb18788.x 3073705

[41] Ilic D Bollinger JM Gelb M Mauro TM. Spla2 and the epidermal barrier. Biochim Biophys Acta (2014) 1841(3):416–21. doi: 10.1016/j.bbalip.2013.11.002 PMC4548940 24269828

[42] Joo KM Hwang JH Bae S Nahm DH Park HS Ye YM. Relationship of ceramide-, and free fatty acid-cholesterol ratios in the stratum corneum with skin barrier function of normal, atopic dermatitis lesional and non-lesional skins. J Dermatol Sci (2015) 77(1):71–4. doi: 10.1016/j.jdermsci.2014.10.001 25455137

[43] Feingold KR Elias PM. Role of lipids in the formation and maintenance of the cutaneous permeability barrier. Biochim Biophys Acta (2014) 1841(3):280–94. doi: 10.1016/j.bbalip.2013.11.007 24262790

[44] Holleran WM Takagi Y Uchida Y. Epidermal sphingolipids: metabolism, function, and roles in skin disorders. FEBS Lett (2006) 580(23):5456–66. doi: 10.1016/j.febslet.2006.08.039 16962101

[45] Li W Sandhoff R Kono M Zerfas P Hoffmann V Ding BC. Depletion of ceramides with very long chain fatty acids causes defective skin permeability barrier function, and neonatal lethality in elovl4 deficient mice. Int J Biol Sci (2007) 3(2):120–8. doi: 10.7150/ijbs.3.120 PMC1796950 17311087

[46] Opalka L Kovacik A Pullmannova P Maixner J Vavrova K. Effects of omega-o-acylceramide structures and concentrations in healthy and diseased skin barrier lipid membrane models. J Lipid Res (2020) 61(2):219–28. doi: 10.1194/jlr.RA119000420 PMC6997605 31857390

[47] Nakamura N Fujimoto T. Adipose differentiation-related protein has two independent domains for targeting to lipid droplets. Biochem Biophys Res Commun (2003) 306(2):333–8. doi: 10.1016/s0006-291x(03)00979-3 12804567

[48] Russo GL. Dietary n-6 and n-3 polyunsaturated fatty acids: from biochemistry to clinical implications in cardiovascular prevention. Biochem Pharmacol (2009) 77(6):937–46. doi: 10.1016/j.bcp.2008.10.020 19022225

[49] Chapkin RS Ziboh VA Marcelo CL Voorhees JJ. Metabolism of essential fatty acids by human epidermal enzyme preparations: evidence of chain elongation. J Lipid Res (1986) 27(9):945–54. doi: 10.1016/S0022-2275(20)38771-X 3097227

[50] Calder PC. Long-chain fatty acids and inflammation. Proc Nutr Soc (2012) 71(2):284–9. doi: 10.1017/S0029665112000067 22369781

[51] Ziboh VA Miller CC Cho Y. Metabolism of polyunsaturated fatty acids by skin epidermal enzymes: generation of antiinflammatory and antiproliferative metabolites. Am J Clin Nutr (2000) 71(1 Suppl):361S–6S. doi: 10.1093/ajcn/71.1.361s 10617998

[52] Gorjao R Azevedo-Martins AK Rodrigues HG Abdulkader F Arcisio-Miranda M Procopio J. Comparative effects of dha and epa on cell function. Pharmacol Ther (2009) 122(1):56–64. doi: 10.1016/j.pharmthera.2009.01.004 19318040

[53] Hansen HS Jensen B. Essential function of linoleic acid esterified in acylglucosylceramide and acylceramide in maintaining the epidermal water permeability barrier. evidence from feeding studies with oleate, linoleate, arachidonate, columbinate and alpha-linolenate. Biochim Biophys Acta (1985) 834(3):357–63. doi: 10.1016/0005-2760(85)90009-8 3922424

[54] Simard M Julien P Fradette J Pouliot R. Modulation of the lipid profile of reconstructed skin substitutes after essential fatty acid supplementation affects testosterone permeability. Cells (2019) 8(10):1142. doi: 10.3390/cells8101142 PMC6829228 31557890

[55] Elias PM. Lipids and the epidermal permeability barrier. Arch Dermatol Res (1981) 270(1):95–117. doi: 10.1007/BF00417155 6167209

[56] Kendall AC Pilkington SM Massey KA Sassano G Rhodes LE Nicolaou A. Distribution of bioactive lipid mediators in human skin. J Invest Dermatol (2015) 135(6):1510–20. doi: 10.1038/jid.2015.41 25668241

[57] Kendall AC Nicolaou A. Bioactive lipid mediators in skin inflammation and immunity. Prog Lipid Res (2013) 52(1):141–64. doi: 10.1016/j.plipres.2012.10.003 23124022

[58] Morrow JD Harris TM Roberts LJ 2nd. Noncyclooxygenase oxidative formation of a series of novel prostaglandins: analytical ramifications for measurement of eicosanoids. Anal Biochem (1990) 184(1):1–10. doi: 10.1016/0003-2697(90)90002-q 2321745

[59] Dalle Carbonare M Del Giudice E Stecca A Colavito D Fabris M D’Arrigo A. A saturated n-acylethanolamine other than n-palmitoyl ethanolamine with anti-inflammatory properties: a neglected story. J Neuroendocrinol (2008) 20 Suppl 1:26–34. doi: 10.1111/j.1365-2826.2008.01689.x 18426496

[60] Da Silva MS Bilodeau JF Larose J Greffard K Julien P Barbier O. Modulation of the biomarkers of inflammation and oxidative stress by ruminant trans fatty acids and dairy proteins in vascular endothelial cells (huvec). Prostaglandins Leukot Essent Fatty Acids (2017) 126:64–71. doi: 10.1016/j.plefa.2017.09.016 29031397

[61] Bos CL Richel DJ Ritsema T Peppelenbosch MP Versteeg HH. Prostanoids and prostanoid receptors in signal transduction. Int J Biochem Cell Biol (2004) 36(7):1187–205. doi: 10.1016/j.biocel.2003.08.006 15109566

[62] Torocsik D Weise C Gericke J Szegedi A Lucas R Mihaly J. Transcriptomic and lipidomic profiling of eicosanoid/docosanoid signalling in affected and non-affected skin of human atopic dermatitis patients. Exp Dermatol (2019) 28(2):177–89. doi: 10.1111/exd.13867 30575130

[63] Yazid S Norling LV Flower RJ. Anti-inflammatory drugs, eicosanoids and the annexin a1/fpr2 anti-inflammatory system. Prostaglandins Other Lipid Mediat (2012) 98(3-4):94–100. doi: 10.1016/j.prostaglandins.2011.11.005 22123264

[64] Calder PC. Polyunsaturated fatty acids, inflammation, and immunity. Lipids (2001) 36(9):1007–24. doi: 10.1007/s11745-001-0812-7 11724453

[65] Legler DF Krause P Scandella E Singer E Groettrup M. Prostaglandin e2 is generally required for human dendritic cell migration and exerts its effect *via* ep2 and ep4 receptors. J Immunol (2006) 176(2):966–73. doi: 10.4049/jimmunol.176.2.966 16393982

[66] Sandulache VC Parekh A Li-Korotky HS Dohar JE Hebda PA. Prostaglandin e2 differentially modulates human fetal and adult dermal fibroblast migration and contraction: implication for wound healing. Wound Repair Regener (2006) 14(5):633–43. doi: 10.1111/j.1743-6109.2006.00156.x 17014677

[67] Camp R Greaves MW. The catabolism of prostaglandins by rat skin. Biochem J (1980) 186(1):153–60. doi: 10.1042/bj1860153 PMC1161514 7370004

[68] von Euler US. On the specific vaso-dilating and plain muscle stimulating substances from accessory genital glands in man and certain animals (prostaglandin and vesiglandin). J Physiol (1936) 88(2):213–34. doi: 10.1113/jphysiol.1936.sp003433 PMC1395258 16994817

[69] Jonsson CE Anggard E. Biosynthesis and metabolism of prostaglandin e 2 in human skin. Scand J Clin Lab Invest (1972) 29(3):289–96. doi: 10.3109/00365517209080244 5037626

[70] Hohjoh H Inazumi T Tsuchiya S Sugimoto Y. Prostanoid receptors and acute inflammation in skin. Biochimie (2014) 107 Pt A:78–81. doi: 10.1016/j.biochi.2014.08.010 25179301

[71] Shim JH. Prostaglandin e2 induces skin aging *via* e-prostanoid 1 in normal human dermal fibroblasts. Int J Mol Sci (2019) 20(22):5555. doi: 10.3390/ijms20225555 PMC6887779 31703303

[72] Kabashima K Sakata D Nagamachi M Miyachi Y Inaba K Narumiya S. Prostaglandin e2-ep4 signaling initiates skin immune responses by promoting migration and maturation of langerhans cells. Nat Med (2003) 9(6):744–9. doi: 10.1038/nm872 12740571

[73] Ziboh VA. The significance of polyunsaturated fatty acids in cutaneous biology. Lipids (1996) 31 Suppl:S249–53. doi: 10.1007/BF02637085 8729128

[74] Burger F Krieg P Marks F Furstenberger G. Positional- and stereo-selectivity of fatty acid oxygenation catalysed by mouse (12s)-lipoxygenase isoenzymes. Biochem J (2000) 348 Pt 2:329–35. doi: 10.1042/bj3480329 PMC1221070 10816426

[75] Schweiger D Furstenberger G Krieg P. Inducible expression of 15-lipoxygenase-2 and 8-lipoxygenase inhibits cell growth *via* common signaling pathways. J Lipid Res (2007) 48(3):553–64. doi: 10.1194/jlr.M600311-JLR200 17164225

[76] Laneuville O Breuer DK Xu N Huang ZH Gage DA Watson JT. Fatty acid substrate specificities of human prostaglandin-endoperoxide h synthase-1 and -2. formation of 12-hydroxy-(9z, 13e/z, 15z)- octadecatrienoic acids from alpha-linolenic acid. J Biol Chem (1995) 270(33):19330–6. doi: 10.1074/jbc.270.33.19330 7642610

[77] Thuresson ED Lakkides KM Smith WL. Different catalytically competent arrangements of arachidonic acid within the cyclooxygenase active site of prostaglandin endoperoxide h synthase-1 lead to the formation of different oxygenated products. J Biol Chem (2000) 275(12):8501–7. doi: 10.1074/jbc.275.12.8501 10722687

[78] Perez B Dahlgaard SE Bulsara P Rawlings AV Jensen MM Dong M. Synthesis and characterization of o-acylated-omega-hydroxy fatty acids as skin-protecting barrier lipids. J Colloid Interface Sci (2017) 490:137–46. doi: 10.1016/j.jcis.2016.11.031 27870954

[79] Sadik CD Sezin T Kim ND. Leukotrienes orchestrating allergic skin inflammation. Exp Dermatol (2013) 22(11):705–9. doi: 10.1111/exd.12239 24433180

[80] Simard-Bisson C Parent LA Moulin VJ Fruteau de Laclos B. Characterization of epidermal lipoxygenase expression in normal human skin and tissue-engineered skin substitutes. J Histochem Cytochem (2018) 66(11):813–24. doi: 10.1369/0022155418788117 PMC6213569 29985723

[81] Cantieri JS Graff G Goldberg ND. Cyclic gmp metabolism in psoriasis: activation of soluble epidermal guanylate cyclase by arachidonic acid and 12-hydroxy-5,8,10,14-eicosatetraenoic acid. J Invest Dermatol (1980) 74(4):234–7. doi: 10.1111/1523-1747.ep12541785 6103015

[82] Dowd PM Kobza Black A Woollard PM Camp RD Greaves MW. Cutaneous responses to 12-hydroxy-5,8,10,14-eicosatetraenoic acid (12-hete). J Invest Dermatol (1985) 84(6):537–41. doi: 10.1111/1523-1747.ep12273537 3998504

[83] Tang DG Chen YQ Honn KV. Arachidonate lipoxygenases as essential regulators of cell survival and apoptosis. Proc Natl Acad Sci USA (1996) 93(11):5241–6. doi: 10.1073/pnas.93.11.5241 PMC39229 8643560

[84] Simard M Grenier A Rioux G Tremblay A Blais I Flamand N. Remodeling of the dermal extracellular matrix in a tissue-engineered psoriatic skin model by n-3 polyunsaturated fatty acids. Biomedicines (2022) 10(5):1078. doi: 10.3390/biomedicines10051078 35625817 PMC9138383

[85] Kragballe K Pinnamaneni G Desjarlais L Duell EA Voorhees JJ. Dermis-derived 15-hydroxy-eicosatetraenoic acid inhibits epidermal 12-lipoxygenase activity. J Invest Dermatol (1986) 87(4):494–8. doi: 10.1111/1523-1747.ep12455564 3093592

[86] Nugteren DH Kivits GA. Conversion of linoleic acid and arachidonic acid by skin epidermal lipoxygenases. Biochim Biophys Acta (1987) 921(1):135–41. doi: 10.1016/0005-2760(87)90179-2 3113487

[87] McDonnell M Davis W Li H Funk CD. Characterization of the murine epidermal 12/15-lipoxygenase. Prostaglandins Other Lipid Mediators (2001) 63(3):93–107. doi: 10.1016/s0090-6980(00)00100-3 11204741

[88] Kowal-Bielecka O Distler O Neidhart M Kunzler P Rethage J Nawrath M. Evidence of 5-lipoxygenase overexpression in the skin of patients with systemic sclerosis: a newly identified pathway to skin inflammation in systemic sclerosis. Arthritis Rheum (2001) 44(8):1865–75. doi: 10.1002/1529-0131(200108)44:8<1865::AID-ART325>3.0.CO;2-M 11508440

[89] Bayer M Mosandl A Thaci D. Improved enantioselective analysis of polyunsaturated hydroxy fatty acids in psoriatic skin scales using high-performance liquid chromatography. J Chromatogr B Analytical Technol Biomed Life Sci (2005) 819(2):323–8. doi: 10.1016/j.jchromb.2005.02.008 15833297

[90] Simard M Tremblay A Morin S Martin C Julien P Fradette J. Alpha-linolenic acid and linoleic acid modulate the lipidome and the skin barrier of a tissue-engineered skin model. Acta biomaterialia (2021) 140:261–74. doi: 10.1016/j.actbio.2021.11.021 34808417

[91] Gron B Iversen L Ziboh V Kragballe K. Distribution of monohydroxy fatty acids in specific human epidermal phospholipids. Exp Dermatol (1993) 2(1):38–44. doi: 10.1111/j.1600-0625.1993.tb00197.x 8156169

[92] Godessart N Camacho M Lopez-Belmonte J Anton R Garcia M de Moragas JM. Prostaglandin h-synthase-2 is the main enzyme involved in the biosynthesis of octadecanoids from linoleic acid in human dermal fibroblasts stimulated with interleukin-1beta. J Invest Dermatol (1996) 107(5):726–32. doi: 10.1111/1523-1747.ep12365616 8875957

[93] Kim C Ryu HC Kim JH. Low-dose uvb irradiation stimulates matrix metalloproteinase-1 expression *via* a blt2-linked pathway in hacat cells. Exp Mol Med (2010) 42(12):833–41. doi: 10.3858/emm.2010.42.12.086 PMC3015157 20966635

[94] Luo L Tanaka R Kanazawa S Lu F Hayashi A Yokomizo T. A synthetic leukotriene b4 receptor type 2 agonist accelerates the cutaneous wound healing process in diabetic rats by indirect stimulation of fibroblasts and direct stimulation of keratinocytes. J Diabetes Complications (2017) 31(1):13–20. doi: 10.1016/j.jdiacomp.2016.09.002 27742551

[95] Levy BD Clish CB Schmidt B Gronert K Serhan CN. Lipid mediator class switching during acute inflammation: signals in resolution. Nat Immunol (2001) 2(7):612–9. doi: 10.1038/89759 11429545

[96] Arita M Bianchini F Aliberti J Sher A Chiang N Hong S. Stereochemical assignment, antiinflammatory properties, and receptor for the omega-3 lipid mediator resolvin E1. J Exp Med (2005) 201(5):713–22. doi: 10.1084/jem.20042031 PMC2212834 15753205

[97] Serhan CN Clish CB Brannon J Colgan SP Chiang N Gronert K. Novel functional sets of lipid-derived mediators with antiinflammatory actions generated from omega-3 fatty acids *via* cyclooxygenase 2-nonsteroidal antiinflammatory drugs and transcellular processing. J Exp Med (2000) 192(8):1197–204. doi: 10.1084/jem.192.8.1197 PMC2195872 11034610

[98] Serhan CN Chiang N Dalli J. The resolution code of acute inflammation: novel pro-resolving lipid mediators in resolution. Semin Immunol (2015) 27(3):200–15. doi: 10.1016/j.smim.2015.03.004 PMC4515371 25857211

[99] Hong S Gronert K Devchand PR Moussignac RL Serhan CN. Novel docosatrienes and 17s-resolvins generated from docosahexaenoic acid in murine brain, human blood, and glial cells. autacoids in anti-inflammation. J Biol Chem (2003) 278(17):14677–87. doi: 10.1074/jbc.M300218200 12590139

[100] Krishnamoorthy S Recchiuti A Chiang N Yacoubian S Lee CH Yang R. Resolvin d1 binds human phagocytes with evidence for proresolving receptors. Proc Natl Acad Sci United States America (2010) 107(4):1660–5. doi: 10.1073/pnas.0907342107 PMC2824371 20080636

[101] Arita M Ohira T Sun YP Elangovan S Chiang N Serhan CN. Resolvin e1 selectively interacts with leukotriene b4 receptor blt1 and chemr23 to regulate inflammation. J Immunol (2007) 178(6):3912–7. doi: 10.4049/jimmunol.178.6.3912 17339491

[102] Serhan CN Levy BD. Resolvins in inflammation: emergence of the pro-resolving superfamily of mediators. J Clin Invest (2018) 128(7):2657–69. doi: 10.1172/JCI97943 PMC6025982 29757195

[103] Chiang N Dalli J Colas RA Serhan CN. Identification of resolvin d2 receptor mediating resolution of infections and organ protection. J Exp Med (2015) 212(8):1203–17. doi: 10.1084/jem.20150225 PMC4516788 26195725

[104] Chiang N Serhan CN. Structural elucidation and physiologic functions of specialized pro-resolving mediators and their receptors. Mol Aspects Med (2017) 58:114–29. doi: 10.1016/j.mam.2017.03.005 PMC5623601 28336292

[105] Serhan CN Krishnamoorthy S Recchiuti A Chiang N. Novel anti-inflammatory–pro-resolving mediators and their receptors. Curr Top Med Chem (2011) 11(6):629–47. doi: 10.2174/1568026611109060629 PMC3094721 21261595

[106] Kendall AC Pilkington SM Murphy SA Del Carratore F Sunarwidhi AL Kiezel-Tsugunova M. Dynamics of the human skin mediator lipidome in response to dietary omega-3 fatty acid supplementation. FASEB journal: Off Publ Fed Am Societies Exp Biol (2019) 33(11):13014–27. doi: 10.1096/fj.201901501R PMC6902719 31518521

[107] Hellmann J Sansbury BE Wong B Li X Singh M Nuutila K. Biosynthesis of d-series resolvins in skin provides insights into their role in tissue repair. J Invest Dermatol (2018) 138(9):2051–60. doi: 10.1016/j.jid.2018.03.1498 PMC6109422 29559341

[108] Munro S Thomas KL Abu-Shaar M. Molecular characterization of a peripheral receptor for cannabinoids. Nature (1993) 365(6441):61–5. doi: 10.1038/365061a0 7689702

[109] Howlett AC Barth F Bonner TI Cabral G Casellas P Devane WA. International union of pharmacology. xxvii. classification of cannabinoid receptors. Pharmacol Rev (2002) 54(2):161–202. doi: 10.1124/pr.54.2.161 12037135

[110] Di Marzo V Deutsch DG. Biochemistry of the endogenous ligands of cannabinoid receptors. Neurobiol Dis (1998) 5(6 Pt B):386–404. doi: 10.1006/nbdi.1998.0214 9974173

[111] Matsuda LA Lolait SJ Brownstein MJ Young AC Bonner TI. Structure of a cannabinoid receptor and functional expression of the cloned cdna. Nature (1990) 346(6284):561–4. doi: 10.1038/346561a0 2165569

[112] Di Marzo V. ‘Endocannabinoids’ and other fatty acid derivatives with cannabimimetic properties: biochemistry and possible physiopathological relevance. Biochim Biophys Acta (1998) 1392(2-3):153–75. doi: 10.1016/s0005-2760(98)00042-3 9630590

[113] McDougle DR Watson JE Abdeen AA Adili R Caputo MP Krapf JE. Anti-inflammatory omega-3 endocannabinoid epoxides. Proc Natl Acad Sci United States America (2017) 114(30):E6034–E43. doi: 10.1073/pnas.1610325114 PMC5544256 28687674

[114] Manca C Shen M Boubertakh B Martin C Flamand N Silvestri C. Alterations of brain endocannabinoidome signaling in germ-free mice. Biochim Biophys Acta Mol Cell Biol Lipids (2020) 1865(12):158786. doi: 10.1016/j.bbalip.2020.158786 32795503

[115] Piscitelli F Silvestri C. Role of the endocannabinoidome in human and mouse atherosclerosis. Curr Pharm Des (2019) 25(29):3147–64. doi: 10.2174/1381612825666190826162735 31448709

[116] Ezzili C Otrubova K Boger DL. Fatty acid amide signaling molecules. Bioorg Med Chem Lett (2010) 20(20):5959–68. doi: 10.1016/j.bmcl.2010.08.048 PMC2942981 20817522

[117] Stander S Schmelz M Metze D Luger T Rukwied R. Distribution of cannabinoid receptor 1 (cb1) and 2 (cb2) on sensory nerve fibers and adnexal structures in human skin. J Dermatol Sci (2005) 38(3):177–88. doi: 10.1016/j.jdermsci.2005.01.007 15927811

[118] Pucci M Pirazzi V Pasquariello N Maccarrone M. Endocannabinoid signaling and epidermal differentiation. Eur J dermatology: EJD (2011) 21 Suppl 2:29–34. doi: 10.1684/ejd.2011.1266 21628127

[119] Gaffal E Glodde N Jakobs M Bald T Tuting T. Cannabinoid 1 receptors in keratinocytes attenuate fluorescein isothiocyanate-induced mouse atopic-like dermatitis. Exp Dermatol (2014) 23(6):401–6. doi: 10.1111/exd.12414 24750433

[120] Pertwee RG Howlett AC Abood ME Alexander SP Di Marzo V Elphick MR. International union of basic and clinical pharmacology. lxxix. cannabinoid receptors and their ligands: beyond cb(1) and cb(2). Pharmacol Rev (2010) 62(4):588–631. doi: 10.1124/pr.110.003004 PMC2993256 21079038

[121] Maccarrone M Di Rienzo M Battista N Gasperi V Guerrieri P Rossi A. The endocannabinoid system in human keratinocytes. evidence that anandamide inhibits epidermal differentiation through cb1 receptor-dependent inhibition of protein kinase c, activation protein-1, and transglutaminase. J Biol Chem (2003) 278(36):33896–903. doi: 10.1074/jbc.M303994200 12815050

[122] McPartland JM. Expression of the endocannabinoid system in fibroblasts and myofascial tissues. J Bodyw Mov Ther (2008) 12(2):169–82. doi: 10.1016/j.jbmt.2008.01.004 19083670

[123] Kendall AC Pilkington SM Sassano G Rhodes LE Nicolaou A. N-acyl ethanolamide and eicosanoid involvement in irritant dermatitis. Br J Dermatol (2016) 175(1):163–71. doi: 10.1111/bjd.14521 26947140

[124] Tinto F Archambault AS Dumais E Rakotoarivelo V Kostrzewa M Martin C. Synthesis and molecular targets of n-13-hydroxy-octadienoyl-ethanolamine, a novel endogenous bioactive 15-lipoxygenase-derived metabolite of n-linoleoyl-ethanolamine found in the skin and saliva. Biochim Biophys Acta Mol Cell Biol Lipids (2021) 1866(8):158954. doi: 10.1016/j.bbalip.2021.158954 33915294

[125] Archambault AS Tinto F Dumais E Rakotoarivelo V Kostrzewa M Plante PL. Biosynthesis of the novel endogenous 15-lipoxygenase metabolites n-13-hydroxy-octodecadienoyl-ethanolamine and 13-hydroxy-octodecadienoyl-glycerol by human neutrophils and eosinophils. Cells (2021) 10(9):2322. doi: 10.3390/cells10092322 34571971 PMC8470279

[126] Dobrosi N Toth BI Nagy G Dozsa A Geczy T Nagy L. Endocannabinoids enhance lipid synthesis and apoptosis of human sebocytes *via* cannabinoid receptor-2-mediated signaling. FASEB J (2008) 22(10):3685–95. doi: 10.1096/fj.07-104877 18596221

[127] Simard M Rakotoarivelo V Di Marzo V Flamand N. Expression and functions of the cb2 receptor in human leukocytes. Front Pharmacol (2022) 13:826400. doi: 10.3389/fphar.2022.826400 35273503 PMC8902156

[128] Facer P Casula MA Smith GD Benham CD Chessell IP Bountra C. Differential expression of the capsaicin receptor trpv1 and related novel receptors trpv3, trpv4 and trpm8 in normal human tissues and changes in traumatic and diabetic neuropathy. BMC Neurol (2007) 7:11. doi: 10.1186/1471-2377-7-11 17521436 PMC1892784

[129] Toth BI Dobrosi N Dajnoki A Czifra G Olah A Szollosi AG. Endocannabinoids modulate human epidermal keratinocyte proliferation and survival *via* the sequential engagement of cannabinoid receptor-1 and transient receptor potential vanilloid-1. J Invest Dermatol (2011) 131(5):1095–104. doi: 10.1038/jid.2010.421 21248768

[130] Lowes MA Bowcock AM Krueger JG. Pathogenesis and therapy of psoriasis. Nature (2007) 445(7130):866–73. doi: 10.1038/nature05663 17314973

[131] Schon MP Boehncke WH. Psoriasis. N Engl J Med (2005) 352(18):1899–912. doi: 10.1056/NEJMra041320 15872205

[132] Greb JE Goldminz AM Elder JT Lebwohl MG Gladman DD Wu JJ. Psoriasis. Nat Rev Dis Primers (2016) 2:16082. doi: 10.1038/nrdp.2016.82 27883001

[133] Mak RK Hundhausen C Nestle FO. Progress in understanding the immunopathogenesis of psoriasis. Actas Dermosifiliogr (2009) 100 Suppl 2:2–13. doi: 10.1016/s0001-7310(09)73372-1 PMC2957885 20096156

[134] Cingoz K Gunduz K Inanir I. Patients’ knowledge about psoriasis and comorbidities; their participation in treatment decisions. J Dermatolog Treat (2021) 32(2):212–4. doi: 10.1080/09546634.2019.1638880 31257954

[135] Knuckles MLF Levi E Soung J. Defining and treating moderate plaque psoriasis: a dermatologist survey. J Dermatolog Treat (2018) 29(7):658–63. doi: 10.1080/09546634.2018.1443200 29502473

[136] Morin A Simard M Rioux G Grenier A Morin S Pouliot R. Application of an *in vitro* psoriatic skin model to study cutaneous metabolization of tazarotene. Processes (2019) 7(12):871. doi: 10.3390/pr7120871

[137] Korver JE van Duijnhoven MW Pasch MC van Erp PE van de Kerkhof PC. Assessment of epidermal subpopulations and proliferation in healthy skin, symptomless and lesional skin of spreading psoriasis. Br J Dermatol (2006) 155(4):688–94. doi: 10.1111/j.1365-2133.2006.07403.x 16965416

[138] Sandby-Moller J Poulsen T Wulf HC. Epidermal thickness at different body sites: relationship to age, gender, pigmentation, blood content, skin type and smoking habits. Acta dermato-venereologica (2003) 83(6):410–3. doi: 10.1080/00015550310015419 14690333

[139] Bernard BA Robinson SM Vandaele S Mansbridge JN Darmon M. Abnormal maturation pathway of keratinocytes in psoriatic skin. Br J Dermatol (1985) 112(6):647–53. doi: 10.1111/j.1365-2133.1985.tb02332.x 3890921

[140] Iizuka H Takahashi H Honma M Ishida-Yamamoto A. Unique keratinization process in psoriasis: late differentiation markers are abolished because of the premature cell death. J Dermatol (2004) 31(4):271–6. doi: 10.1111/j.1346-8138.2004.tb00672.x 15187321

[141] Greb JE Goldminz AM Gottlieb AB. Insights on methotrexate in psoriatic disease. Clin Immunol (2016) 172:61–4. doi: 10.1016/j.clim.2016.07.008 27455859

[142] Lorthois I Simard M Morin S Pouliot R. Infiltration of t cells into a three-dimensional psoriatic skin model mimics pathological key features. Int J Mol Sci (2019) 20(7):1670. doi: 10.3390/ijms20071670 PMC6479293 30987186

[143] Rioux G Simard M Morin S Lorthois I Guerin SL Pouliot R. Development of a 3d psoriatic skin model optimized for infiltration of il-17a producing t cells: focus on the crosstalk between t cells and psoriatic keratinocytes. Acta biomaterialia (2021) 136:210–22. doi: 10.1016/j.actbio.2021.09.018 34547515

[144] Benhadou F Mintoff D Del Marmol V. Psoriasis: keratinocytes or immune cells - which is the trigger? Dermatology (2019) 235(2):91–100. doi: 10.1159/000495291 30566935

[145] Krueger JG Bowcock A. Psoriasis pathophysiology: current concepts of pathogenesis. Ann rheumatic Dis (2005) 64 Suppl 2:ii30–6. doi: 10.1136/ard.2004.031120 PMC1766865 15708932

[146] Lynde CW Poulin Y Vender R Bourcier M Khalil S. Interleukin 17a: toward a new understanding of psoriasis pathogenesis. J Am Acad Dermatol (2014) 71(1):141–50. doi: 10.1016/j.jaad.2013.12.036 24655820

[147] Boniface K Bernard FX Garcia M Gurney AL Lecron JC Morel F. Il-22 inhibits epidermal differentiation and induces proinflammatory gene expression and migration of human keratinocytes. J Immunol (2005) 174(6):3695–702. doi: 10.4049/jimmunol.174.6.3695 15749908

[148] Liang J Chen P Li C Li D Wang J Xue R. Il-22 down-regulates cx43 expression and decreases gap junctional intercellular communication by activating the jnk pathway in psoriasis. J Invest Dermatol (2019) 139(2):400–11. doi: 10.1016/j.jid.2018.07.032 30171832

[149] Motta S Monti M Sesana S Caputo R Carelli S Ghidoni R. Ceramide composition of the psoriatic scale. Biochim Biophys Acta (1993) 1182(2):147–51. doi: 10.1016/0925-4439(93)90135-n 8357845

[150] Baer AN Klaus MV Green FA. Epidermal fatty acid oxygenases are activated in non-psoriatic dermatoses. J Invest Dermatol (1995) 104(2):251–5. doi: 10.1111/1523-1747.ep12612793 7829882

[151] Wojcik P Biernacki M Wronski A Luczaj W Waeg G Zarkovic N. Altered lipid metabolism in blood mononuclear cells of psoriatic patients indicates differential changes in psoriasis vulgaris and psoriatic arthritis. Int J Mol Sci (2019) 20(17):4249. doi: 10.3390/ijms20174249 PMC6747546 31480263

[152] Kassis V Weismann K Heiligstadt H Sondergaard J. Synthesis of prostaglandins by psoriatic skin. Arch Dermatol Res (1977) 259(3):207–12. doi: 10.1007/BF00561448 199118

[153] Honda T Kabashima K. Prostanoids and leukotrienes in the pathophysiology of atopic dermatitis and psoriasis. Int Immunol (2019) 31(9):589–95. doi: 10.1093/intimm/dxy087 30715370

[154] Sorokin AV Norris PC English JT Dey AK Chaturvedi A Baumer Y. Identification of proresolving and inflammatory lipid mediators in human psoriasis. J Clin lipidology (2018) 12(4):1047–60. doi: 10.1016/j.jacl.2018.03.091 PMC6112609 29730187

[155] Scher JU Pillinger MH. 15d-Pgj2: the anti-inflammatory prostaglandin? Clin Immunol (2005) 114(2):100–9. doi: 10.1016/j.clim.2004.09.008 15639643

[156] Powell WS Rokach J. Biosynthesis, biological effects, and receptors of hydroxyeicosatetraenoic acids (hetes) and oxoeicosatetraenoic acids (oxo-etes) derived from arachidonic acid. Biochim Biophys Acta (2015) 1851(4):340–55. doi: 10.1016/j.bbalip.2014.10.008 PMC5710736 25449650

[157] Fogh K Sogaard H Herlin T Kragballe K. Improvement of psoriasis vulgaris after intralesional injections of 15-hydroxyeicosatetraenoic acid (15-hete). J Am Acad Dermatol (1988) 18(2 Pt 1):279–85. doi: 10.1016/s0190-9622(88)70040-7 3346412

[158] Yoo H Jeon B Jeon MS Lee H Kim TY. Reciprocal regulation of 12- and 15-lipoxygenases by uv-irradiation in human keratinocytes. FEBS Lett (2008) 582(21-22):3249–53. doi: 10.1016/j.febslet.2008.08.017 18755188

[159] Sapieha P Stahl A Chen J Seaward MR Willett KL Krah NM. 5-lipoxygenase metabolite 4-hdha is a mediator of the antiangiogenic effect of omega-3 polyunsaturated fatty acids. Sci Transl Med (2011) 3(69):69ra12. doi: 10.1126/scitranslmed.3001571 PMC3711031 21307302

[160] Ikai K. Psoriasis and the arachidonic acid cascade. J Dermatol Sci (1999) 21(3):135–46. doi: 10.1016/s0923-1811(99)00042-0 10527374

[161] Evans JF. Cysteinyl leukotriene receptors. Prostaglandins Other Lipid Mediat (2002) 68-69:587–97. doi: 10.1016/s0090-6980(02)00057-6 12432945

[162] Fogh K Herlin T Kragballe K. Eicosanoids in acute and chronic psoriatic lesions: leukotriene b4, but not 12-hydroxy-eicosatetraenoic acid, is present in biologically active amounts in acute guttate lesions. J Invest Dermatol (1989) 92(6):837–41. doi: 10.1111/1523-1747.ep12696858 2542417

[163] Leiria LO Wang CH Lynes MD Yang K Shamsi F Sato M. 12-lipoxygenase regulates cold adaptation and glucose metabolism by producing the omega-3 lipid 12-hepe from brown fat. Cell Metab (2019) 30(4):768–83 e7. doi: 10.1016/j.cmet.2019.07.001 PMC6774888 31353262

[164] Saika A Nagatake T Hirata SI Sawane K Adachi J Abe Y. Omega3 fatty acid metabolite, 12-hydroxyeicosapentaenoic acid, alleviates contact hypersensitivity by downregulation of cxcl1 and cxcl2 gene expression in keratinocytes *via* retinoid x receptor alpha. FASEB journal: Off Publ Fed Am Societies Exp Biol (2021) 35(4):e21354. doi: 10.1096/fj.202001687R 33749892

[165] Sawane K Nagatake T Hosomi K Hirata SI Adachi J Abe Y. Dietary omega-3 fatty acid dampens allergic rhinitis *via* eosinophilic production of the anti-allergic lipid mediator 15-hydroxyeicosapentaenoic acid in mice. Nutrients (2019) 11(12):2868. doi: 10.3390/nu11122868 PMC6950470 31766714

[166] Li J Chen CY Arita M Kim K Li X Zhang H. An omega-3 polyunsaturated fatty acid derivative, 18-hepe, protects against cxcr4-associated melanoma metastasis. Carcinogenesis (2018) 39(11):1380–8. doi: 10.1093/carcin/bgy117 PMC7191087 30184109

[167] Barr RM Wong E Mallet AI Olins LA Greaves MW. The analysis of arachidonic acid metabolites in normal, uninvolved and lesional psoriatic skin. Prostaglandins (1984) 28(1):57–65. doi: 10.1016/0090-6980(84)90113-8 6435188

[168] Bacon KB Camp RDR. Lipid lymphocyte chemoattractants in psoriasis. Prostaglandins (1990) 40(6):603–14. doi: 10.1016/0090-6980(90)90005-g 2093937

[169] Hein R Gross E Ruzicka T Krieg T. 12-hydroxyeicosatetraenoic acid (12-hete) is a chemotactic stimulus for epidermal cells. Arch Dermatol Res (1991) 283(2):135–7. doi: 10.1007/BF00371624 2069413

[170] Cook-Moreau JM El-Makhour Hojeij Y Barriere G Rabinovitch-Chable HC Faucher KS Sturtz FG. Expression of 5-lipoxygenase (5-lox) in t lymphocytes. Immunology (2007) 122(2):157–66. doi: 10.1111/j.1365-2567.2007.02621.x PMC2265994 17484769

[171] Oi N Yamamoto H Langfald A Bai R Lee MH Bode AM. Lta4h regulates cell cycle and skin carcinogenesis. Carcinogenesis (2017) 38(7):728–37. doi: 10.1093/carcin/bgx049 PMC6248358 28575166

[172] Sumida H Yanagida K Kita Y Abe J Matsushima K Nakamura M. Interplay between cxcr2 and blt1 facilitates neutrophil infiltration and resultant keratinocyte activation in a murine model of imiquimod-induced psoriasis. J Immunol (2014) 192(9):4361–9. doi: 10.4049/jimmunol.1302959 24663678

[173] Sawada Y Honda T Nakamizo S Otsuka A Ogawa N Kobayashi Y. Resolvin E1 attenuates murine psoriatic dermatitis. Sci Rep (2018) 8(1):11873. doi: 10.1038/s41598-018-30373-1 30089836 PMC6082864

[174] Camp R Jones RR Brain S Woollard P Greaves M. Production of intraepidermal microabscesses by topical application of leukotriene b4. J Invest Dermatol (1984) 82(2):202–4. doi: 10.1111/1523-1747.ep12259945 6319504

[175] Hendriks AG Keijsers RR Seyger MM van de Kerkhof PC van Erp PE. Cutaneous application of leukotriene b4 as an *in vivo* model of psoriasis-like skin inflammation: an immunohistological study. Skin Pharmacol Physiol (2014) 27(3):120–6. doi: 10.1159/000354119 24401330

[176] Kragballe K Duell EA Voorhees JJ. Selective decrease of 15-hydroxyeicosatetraenoic acid (15-hete) formation in uninvolved psoriatic dermis. Arch Dermatol (1986) 122(8):877–80. doi:10.1001/archderm.1986.01660200049012 3090943

[177] Tyrrell VJ Ali F Boeglin WE Andrews R Burston J Birchall JC. Lipidomic and transcriptional analysis of the linoleoyl-omega-hydroxyceramide biosynthetic pathway in human psoriatic lesions. J Lipid Res (2021) 62:100094. doi: 10.1016/j.jlr.2021.100094 34171322 PMC8326207

[178] Ruzicka T Simmet T Peskar BA Ring J. Skin levels of arachidonic acid-derived inflammatory mediators and histamine in atopic dermatitis and psoriasis. J Invest Dermatol (1986) 86(2):105–8. doi: 10.1111/1523-1747.ep12284061 3018086

[179] Arai KY Fujioka A Okamura R Nishiyama T. Stimulatory effect of fibroblast-derived prostaglandin e(2) on keratinocyte stratification in the skin equivalent. Wound Repair Regener (2014) 22(6):701–11. doi: 10.1111/wrr.12228 25224163

[180] Sato T Kirimura Y Mori Y. The co-culture of dermal fibroblasts with human epidermal keratinocytes induces increased prostaglandin e2 production and cyclooxygenase 2 activity in fibroblasts. J Invest Dermatol (1997) 109(3):334–9. doi: 10.1111/1523-1747.ep12335935 9284101

[181] Sheibanie AF Tadmori I Jing H Vassiliou E Ganea D. Prostaglandin E2 induces il-23 production in bone marrow-derived dendritic cells. FASEB journal: Off Publ Fed Am Societies Exp Biol (2004) 18(11):1318–20. doi: 10.1096/fj.03-1367fje 15180965

[182] Schirmer C Klein C von Bergen M Simon JC Saalbach A. Human fibroblasts support the expansion of il-17-producing t cells *via* up-regulation of il-23 production by dendritic cells. Blood (2010) 116(10):1715–25. doi: 10.1182/blood-2010-01-263509 20538798

[183] Gao Y Yi X Ding Y. Combined transcriptomic analysis revealed akr1b10 played an important role in psoriasis through the dysregulated lipid pathway and overproliferation of keratinocyte. BioMed Res Int (2017) 2017:8717369. doi: 10.1155/2017/8717369 29204449 PMC5674492

[184] Rioux G Pouliot-Berube C Simard M Benhassine M Soucy J Guerin SL. The tissue-engineered human psoriatic skin substitute: a valuable *in vitro* model to identify genes with altered expression in lesional psoriasis. Int J Mol Sci (2018) 19(10):2923. doi: 10.3390/ijms19102923 PMC6213003 30261611

[185] Rioux G Ridha Z Simard M Turgeon F Guerin SL Pouliot R. Transcriptome profiling analyses in psoriasis: a dynamic contribution of keratinocytes to the pathogenesis. Genes (Basel) (2020) 11(10). doi: 10.3390/genes11101155 PMC7600703 33007857

[186] Guida B Napoleone A Trio R Nastasi A Balato N Laccetti R. Energy-restricted, n-3 polyunsaturated fatty acids-rich diet improves the clinical response to immuno-modulating drugs in obese patients with plaque-type psoriasis: a randomized control clinical trial. Clin Nutr (2014) 33(3):399–405. doi: 10.1016/j.clnu.2013.09.010 24120032

[187] Bittiner SB Tucker WF Cartwright I Double-Blind BSSA. Randomised, placebo-controlled trial of fish oil in psoriasis. Lancet (1988) 1(8582):378–80. doi: 10.1016/s0140-6736(88)91181-6 2893189

[188] Bjorneboe A Smith AK Bjorneboe GE Thune PO Drevon CA. Effect of dietary supplementation with n-3 fatty acids on clinical manifestations of psoriasis. Br J Dermatol (1988) 118(1):77–83. doi: 10.1111/j.1365-2133.1988.tb01753.x 2829958

[189] Escobar SO Achenbach R Iannantuono R Torem V. Topical fish oil in psoriasis–a controlled and blind study. Clin Exp Dermatol (1992) 17(3):159–62. doi: 10.1111/j.1365-2230.1992.tb00194.x 1451289

[190] Grimminger F Mayser P Papavassilis C Thomas M Schlotzer E Heuer KU. A double-blind, randomized, placebo-controlled trial of n-3 fatty acid based lipid infusion in acute, extended guttate psoriasis. rapid improvement of clinical manifestations and changes in neutrophil leukotriene profile. Clin Investig (1993) 71(8):634–43. doi: 10.1007/BF00184491 8219661

[191] Soyland E Funk J Rajka G Sandberg M Thune P Rustad L. Effect of dietary supplementation with very-long-chain n-3 fatty acids in patients with psoriasis. N Engl J Med (1993) 328(25):1812–6. doi: 10.1056/NEJM199306243282504 8502270

[192] Veale DJ Torley HI Richards IM O’Dowd A Fitzsimons C Belch JJ. A double-blind placebo controlled trial of efamol marine on skin and joint symptoms of psoriatic arthritis. Br J Rheumatol (1994) 33(10):954–8. doi: 10.1093/rheumatology/33.10.954 7921757

[193] Balbas GM Regana MS Millet PU. Study on the use of omega-3 fatty acids as a therapeutic supplement in treatment of psoriasis. Clin Cosmet Investig Dermatol (2011) 4:73–7. doi: 10.2147/CCID.S17220 PMC3133503 21760742

[194] Adil M Singh PK Maheshwari K. Clinical evaluation of omega-3 fatty acids in psoriasis. Dermatol Rev (2017) 3:314–23. doi: 10.5114/dr.2017.68778

[195] Saito-Sasaki N Sawada Y Mashima E Yamaguchi T Ohmori S Yoshioka H. Maresin-1 suppresses imiquimod-induced skin inflammation by regulating il-23 receptor expression. Sci Rep (2018) 8(1):5522. doi: 10.1038/s41598-018-23623-9 29615641 PMC5882824

[196] Park KD Kim N Kang J Dhakal H Kim JY Jang YH. Protectin d1 reduces imiquimod-induced psoriasiform skin inflammation. Int Immunopharmacol (2021) 98:107883. doi: 10.1016/j.intimp.2021.107883 34153674

[197] Xu J Duan X Hu F Poorun D Liu X Wang X. Resolvin d1 attenuates imiquimod-induced mice psoriasiform dermatitis through mapks and nf-kappab pathways. J Dermatol Sci (2018) 89(2):127–35. doi: 10.1016/j.jdermsci.2017.10.016 29137840

[198] Lee SH Tonello R Im ST Jeon H Park J Ford Z. Resolvin d3 controls mouse and human trpv1-positive neurons and preclinical progression of psoriasis. Theranostics (2020) 10(26):12111–26. doi: 10.7150/thno.52135 PMC7667671 33204332

[199] Simard M Rioux G Morin S Martin C Guerin SL Flamand N. Investigation of omega-3 polyunsaturated fatty acid biological activity in a tissue-engineered skin model involving psoriatic cells. J Invest Dermatol (2021) 141(10):2391–2401.e13. doi: 10.1016/j.jid.2021.02.755 33857488

[200] Morin S Simard M Rioux G Julien P Pouliot R. Alpha-linolenic acid modulates t cell incorporation in a 3d tissue-engineered psoriatic skin model. Cells (2022) 11(9):1513. doi: 10.3390/cells11091513 35563819 PMC9104007

[201] Morin S Simard M Flamand N Pouliot R. Biological action of docosahexaenoic acid in a 3d tissue-engineered psoriatic skin model: focus on the ppar signaling pathway. Biochim Biophys Acta Mol Cell Biol Lipids (2021) 1866(12):159032. doi: 10.1016/j.bbalip.2021.159032 34428549

[202] Upala S Yong WC Theparee T Sanguankeo A. Effect of omega-3 fatty acids on disease severity in patients with psoriasis: a systematic review. Int J Rheum Dis (2017) 20(4):442–50. doi: 10.1111/1756-185X.13051 28261950

[203] Qin S Wen J Bai XC Chen TY Zheng RC Zhou GB. Endogenous n-3 polyunsaturated fatty acids protect against imiquimod-induced psoriasis-like inflammation *via* the il-17/il-23 axis. Mol Med Rep (2014) 9(6):2097–104. doi: 10.3892/mmr.2014.2136 PMC4055457 24718773

[204] Yang SJ Chi CC. Effects of fish oil supplement on psoriasis: a meta-analysis of randomized controlled trials. BMC Complement Altern Med (2019) 19(1):354. doi: 10.1186/s12906-019-2777-0 31805911 PMC6896351

[205] Roy B Simard M Lorthois I Bélanger A Maheux M Duque-Fernandez A. *In vitro* models of psoriasis. Skin Tissue Models Regenerative Med Elsevier (2018), 103–28. doi: 10.1016/B978-0-12-810545-0.00005-X

[206] Clark CCT Taghizadeh M Nahavandi M Jafarnejad S. Efficacy of omega-3 supplementation in patients with psoriasis: a meta-analysis of randomized controlled trials. Clin Rheumatol (2019) 38(4):977–88. doi: 10.1007/s10067-019-04456-x 30778861

[207] Jaudszus A Gruen M Watzl B Ness C Roth A Lochner A. Evaluation of suppressive and pro-resolving effects of epa and dha in human primary monocytes and t-helper cells. J Lipid Res (2013) 54(4):923–35. doi: 10.1194/jlr.P031260 PMC3605999 23349208

[208] El-Waseef D. A highlight on cd4(+) t-cells in the spleen in a rat model of rheumatoid arthritis and possible therapeutic effect of omega-3. histological and immunofluorescence study. Int Immunopharmacol (2020) 81:106283. doi: 10.1016/j.intimp.2020.106283 32044655

[209] Kinsella JE Lokesh B Broughton S Whelan J. Dietary polyunsaturated fatty acids and eicosanoids: potential effects on the modulation of inflammatory and immune cells: an overview. Nutrition (1990) 6(1):24–44. 2135755

[210] Gorjao R Hirabara SM de Lima TM Cury-Boaventura MF Curi R. Regulation of interleukin-2 signaling by fatty acids in human lymphocytes. J Lipid Res (2007) 48(9):2009–19. doi: 10.1194/jlr.M700175-JLR200 17592174

[211] Lee J Choi YR Kim M Park JM Kang M Oh J. Common and differential effects of docosahexaenoic acid and eicosapentaenoic acid on helper t-cell responses and associated pathways. BMB Rep (2021) 54(5):278–83. doi: 10.5483/BMBRep.2021.54.5.267 PMC8167247 33972011

[212] Switzer KC Fan YY Wang N McMurray DN Chapkin RS. Dietary n-3 polyunsaturated fatty acids promote activation-induced cell death in th1-polarized murine cd4+ t-cells. J Lipid Res (2004) 45(8):1482–92. doi: 10.1194/jlr.M400028-JLR200 PMC4469998 15145980

[213] Pan Y Tian T Park CO Lofftus SY Mei S Liu X. Survival of tissue-resident memory t cells requires exogenous lipid uptake and metabolism. Nature (2017) 543(7644):252–6. doi: 10.1038/nature21379 PMC5509051 28219080

[214] Honda T Kabashima K. Current understanding of the role of dietary lipids in the pathophysiology of psoriasis. J Dermatol Sci (2019) 94(3):314–20. doi: 10.1016/j.jdermsci.2019.05.003 31133503

[215] Dennis EA Norris PC. Eicosanoid storm in infection and inflammation. Nat Rev Immunol (2015) 15(8):511–23. doi: 10.1038/nri3859 PMC4606863 26139350

[216] Sawada Y Honda T Hanakawa S Nakamizo S Murata T Ueharaguchi-Tanada Y. Resolvin e1 inhibits dendritic cell migration in the skin and attenuates contact hypersensitivity responses. J Exp Med (2015) 212(11):1921–30. doi: 10.1084/jem.20150381 PMC4612099 26438363

[217] Zhang P Wu MX. A clinical review of phototherapy for psoriasis. Lasers Med Sci (2018) 33(1):173–80. doi: 10.1007/s10103-017-2360-1 PMC5756569 29067616

[218] Manolis AA Manolis TA Melita H Manolis AS. Psoriasis and cardiovascular disease: the elusive link. Int Rev Immunol (2019) 38(1):33–54. doi: 10.1080/08830185.2018.1539084 30457023

[219] Yagi S Fukuda D Aihara KI Akaike M Shimabukuro M Sata M. N-3 polyunsaturated fatty acids: promising nutrients for preventing cardiovascular disease. J Atheroscler Thromb (2017) 24(10):999–1010. doi: 10.5551/jat.RV17013 PMC5656772 28835582

[220] Skulas-Ray AC Wilson PWF Harris WS Brinton EA Kris-Etherton PM Richter CK. Omega-3 fatty acids for the management of hypertriglyceridemia: a science advisory from the american heart association. Circulation (2019) 140(12):e673–e91. doi: 10.1161/CIR.0000000000000709 31422671

[221] Lembke P Capodice J Hebert K Swenson T. Influence of omega-3 (n3) index on performance and wellbeing in young adults after heavy eccentric exercise. J Sports Sci Med (2014) 13(1):151–6. PMC3918552 24570619

[222] Harris WS Von Schacky C. The omega-3 index: a new risk factor for death from coronary heart disease? Prev Med (2004) 39(1):212–20. doi: 10.1016/j.ypmed.2004.02.030 15208005

